# Metabolomics and neuroanatomical evaluation of post-mortem changes in the hippocampus

**DOI:** 10.1007/s00429-017-1375-5

**Published:** 2017-03-11

**Authors:** Carolina Gonzalez-Riano, Silvia Tapia-González, Antonia García, Alberto Muñoz, Javier DeFelipe, Coral Barbas

**Affiliations:** 1 CEMBIO (Centre for Metabolomics and Bioanalysis), Facultad de Farmacia, Universidad CEU San Pablo, Campus Monteprincipe, Boadilla del Monte, 28668 Madrid, Spain; 2 Laboratorio Cajal de Circuitos Corticales (CTB), Universidad Politécnica de Madrid, Madrid, Spain; 3 Instituto Cajal (CSIC), Avenida Doctor Arce 37, 28002 Madrid, Spain; 4 Centro de Investigación Biomédica en Red sobre Enfermedades Neurodegenerativas (CIBERNED), ISCIII, Madrid, Spain; 5 Department of Cell Biology, Complutense University, Madrid, Spain

**Keywords:** Post-mortem delay, Brain tissue, Metabolomic changes, Hippocampus, Histology, GABAergic interneurons, GABA, Neuronal markers, SMI-32, GAD-65, Parvalbumin, Calbindin-D28K (CB), NADPH-diaphorase (NADPH-d) glutamatergic axon terminals, Microglia, Astrocytes, Metabolites, Creatine, Glutamate, Arginine, Putrescine, Cadaverine, Aspartic acid, GC–MS analysis, LC–MS analysis, Gangliosides, Phosphatidylcholine and phosphatidylethanolamine, Glycerophosphocholine, Glycerophospholipids, 2-Arachidonoylglycerol; anandamide

## Abstract

Understanding the human brain is the ultimate goal in neuroscience, but this is extremely challenging in part due to the fact that brain tissue obtained from autopsy is practically the only source of normal brain tissue and also since changes at different levels of biological organization (genetic, molecular, biochemical, anatomical) occur after death due to multiple mechanisms. Here we used metabolomic and anatomical techniques to study the possible relationship between post-mortem time (PT)-induced changes that may occur at both the metabolomics and anatomical levels in the same brains. Our experiments have mainly focused on the hippocampus of the mouse. We found significant metabolomic changes at 2 h PT, whereas the integrity of neurons and glia, at the anatomical/ neurochemical level, was not significantly altered during the first 5 h PT for the majority of histological markers.

**Electronic supplementary material:**

The online version of this article (doi:10.1007/s00429-017-1375-5) contains supplementary material, which is available to authorized users.

## Introduction

Brain tissue obtained from autopsy is practically the only source of normal brain tissue that can be used to study the structure of the human brain. In general, the major limitation is the post-mortem time (PT) since the longer the PT delay, the larger are the alterations observed in the measurements at all levels of biological organization (genetic, molecular, biochemical, anatomical). Previous studies with a large variety of techniques have shown alterations in expression levels of structural proteins and enzymes and in the integrity of nucleic acids, as well as changes in the morphology of neurons, receptors for neurotransmitters or their transporters at several PTs (Buell 1982; Hilbig et al. 2004; Williams et al. 1978; Perry et al. 1981; Spokes 1979). In addition, brain fixation and storage time as well as certain antemortem factors (age, sex, use of toxic substances and drugs, duration of agonal state, etc.) are all critical and should be considered as they may affect measurements. Thus, brain banks take special care in trying to provide high-quality human tissue for research by performing rapid autopsies and collecting tissue, including matching for several factors both antemortem and post-mortem (Ravid and Swaab 1993).

Perfused animal brains are the source of tissue in most experimental laboratories. For obvious reasons, in humans, we cannot directly test the differences that may occur between tissue fixed by perfusion with no PT delay and tissue obtained after autopsy. In previous studies, it has been shown that the biopsy material obtained during neurosurgical treatment for epilepsy or for removing certain brain tumors represents an excellent opportunity to study the microanatomy of the human brain because the resected tissue can be immediately immersed in the fixative (Del Rio and DeFelipe 1994; Alonso-Nanclares et al. 2008). Thus, this tissue is lacking possible post-mortem time-induced changes that may occur at both the neurochemical and anatomical levels, which is the major problem when using brain tissue from autopsies. Therefore, this kind of tissue is of great value since, for clear ethical reasons, it is as close to a “normal” sample of brain tissue as is possible to obtain for studying the human brain. The major limitation is that different medical characteristics of the patients (i.e., differences in the medication, severity of the disease, onset and duration) may modify the brain tissue. Furthermore, the major source of biopsy material is from the anterior part of the temporal lobe and, therefore, studies in other regions of the brain are scarce. For these reasons, it is important to define what a good quality of human brain tissue obtained at autopsy is when studying any region of the human brain. In general, at least 1- to 2-h PT delay is the minimum time that can be achieved and indeed this is the exception; in most brain banks, PT is 5–10 or more hours before fixation. This is an important limitation, but, unfortunately, animal experimental data are often extrapolated to the human brain without considering the PT delay factors. For example, it is common to describe similar or different patterns of immunostaining when comparing brain tissue from humans with experimental animals without considering these methodological factors. Nevertheless, some measurements are rather stable for many hours, whereas others may rapidly change in minutes after death. Therefore, it is of great relevance to know which are the most sensitive and reliable markers of tissue quality to analyze aspects of the healthy and diseased brain structure at different levels of biological organization in post-mortem cases.

As described in the literature, the rapidly emerging field of metabolomics has been proven to provide very useful information regarding the biochemical basis of neurological and psychiatric disorders, using different analytical platforms and statistical and multi-variant methods for information extraction and data interpretation (Durrenberger et al. 2010). However, very little is known about the possible relationship between PT-induced changes that may occur at both the metabolomics and anatomical levels in the same brains. In the present study, we have attempted to analyze these changes in the same brains with different PT intervals. We have mainly focused on the hippocampus, as this brain region is the target of numerous studies of major brain diseases such as Alzheimer’s disease and epilepsy. We selected different PT intervals of up to 5 h since, in our experience, after this period fine anatomical tools like intracellular injections in fixed material or electron microscopy techniques do not render satisfactory results, whereas the results are excellent with shorter times (Merino-Serrais et al. 2013; Blazquez-Llorca et al. 2013, 2010). Here we used mass spectrometry coupled to several separation techniques (GC–MS and LC–MS) and a variety of histological, histochemical and immunohistochemical techniques that are commonly used to study the neuroanatomical characteristics of the brain in both health and disease. Furthermore, to find an explanation for the degree of metabolite retention existing at a precise time after death, we tried to interpret the obtained results from a biological point of view, providing insight into the hypoxic and decomposition post-mortem reactions. The examination of the trend of the amino compounds, lipids and related metabolites in hippocampus tissue made it possible to assess some of the changes that occur over time following death (Perry et al. 1981). Finally, we compared the neuroanatomical and neurochemical characteristics of brain tissue obtained after perfusion with immersion-fixed brain tissue that was extracted immediately after death (PT 0 h, 0 min) or after several PT delays up to 5 h.

## Materials and methods

### Animals

This study was performed in 2-month-old male C57BL/6 J mice (Charles River Laboratories, Wilmington, MA). Mice were kept in a 12:12-h light/dark cycle and received food and water *ad libitum*. All experimental protocols involving the use of animals were performed in accordance with recommendations for the proper care and use of laboratory animals and under the authorization of the regulations and policies governing the care and use of laboratory animals [EU directive no. 86/609 and Council of Europe Convention ETS1 23, EU decree 2001-486 and Statement of Compliance with Standards for Use of Laboratory Animals by Foreign Institutions no A5388-01, National Institutes of Health (USA)]. Special care was taken to minimize animal suffering and to reduce the number of animals used to the minimum required for statistical accuracy.

In this study, we used two groups of mice, one prepared for histology and immunohistochemistry and the other for metabolomic assays as follows.

### Anatomy: tissue processing, histology and immunohistochemistry

One group was anaesthetized with a pentobarbital lethal injection (40 mg/kg BW, Vetoquinol, Madrid, Spain) and transcardially perfused with 20 mL of 0.1 mol/L phosphate buffer (PB), followed by 150 mL of 4% paraformaldehyde in PB. The brains were removed from the skull, post-fixed in the same solution for 20 h and cryoprotected in 30% sucrose for 2 days. Finally, the brains were flash-frozen in isopentane (2-methylbutane, Merck, Billerica, MA), cooled in a 70% ethanol-dry ice bath and stored at −80 °C until cutting.

The second group of mice was killed with the same pentobarbital lethal injection as described above. Thereafter, their brains were removed after the following PT intervals: 0′ h (0′ min), 30 min, 1, 2, 3 and 5 h (*n* = 3 animals per interval) as follows: Their bodies were maintained at room temperature (animals with PT delay of 30 min, 1 h, 2 h and 3 h), or 2 h at room temperature followed by 3 h at 4 °C in a fridge (animals with PT of 5 h). The skull was maintained joined to the body until decapitation, after which the brains were removed from the skull and fixed in PB-buffered 4% paraformaldehyde for 20 h overnight at 4 °C. After fixation, brains were cryoprotected and frozen as described previously for the perfused tissue.

The brain tissue from the two groups of animals was cut into 30-μm-thick coronal slices with a freezing sliding microtome (Microm HM 450, Microm International, Germany) and processed for histological, histochemical and immunohistochemical experiments (immunoperoxidase and immunofluorescence). For a comprehensive list of the antibodies used and their characteristics, see Supplementary Table S1.

Histochemical experiments involved staining for nicotine adenine dinucleotide phosphate (NADPH)-diaphorase (NADPH-d) according to the protocol of Hope and Vincent (1989). Briefly, sections were washed in 50 mM Tris–HCl, pH 7.4 and incubated for 60 min at 37 °C in 1 mM β-NADPH (Sigma, St Louis, MO), 0.5 mM nitroblue tetrazolium (NBT; Sigma) and 0.1% Triton X-100 in 50 mM Tris–HCl, pH 8. The sections were then rinsed in 50 mM Tris–HCl, pH 7.4 and washed in PB. The sections were mounted, dehydrated, cleared in xylene and then coverslipped.

Immunoperoxidase stainings were carried out in free-floating sections under moderate shaking. The endogenous peroxidase activity was quenched for 1 h at room temperature in a solution of 2% hydrogen peroxide in 100% methanol. After several washes in 0.1 M phosphate buffer (pH 7.4), containing 0.3% TritonX-100 (washing buffer), sections were incubated overnight at 4 °C with one of the following primary antibodies: anti-NeuN (ABN78, rabbit polyclonal, Millipore, Billerica, MA; diluted 1:2000); anti-calbindin D28K (rabbit polyclonal, Swant, Switzerland; diluted 1:2000); anti-parvalbumin (PV-25, rabbit polyclonal, Swant, Switzerland; diluted 1:2000); anti-SMI-32 (SMI-32R, mouse monoclonal, Covance, Princeton, NY; diluted 1:4000); anti-type 1 vesicular glutamate transporter (vGLUT1) (AB5905, guinea pig polyclonal, Millipore, Billerica, MA; diluted 1:5000); anti-type 2 vesicular glutamate transporter (vGLUT2) (AB2251, guinea pig polyclonal, Millipore, Billerica, MA; diluted 1:2000); anti-vesicular gamma-aminobutyric acid transporter vGAT (131003, rabbit polyclonal, Synaptic systems, Göttingen, Germany; diluted 1:2000); anti-Iba1 (ionized calcium binding adaptor protein 1) (019-19741, Wako, Osaka, Japan; diluted 1:500); anti-GFAP (Glial Fibrillary Acidic Protein) (G9269, rabbit polyclonal, Sigma-Aldrich, St Louis. MO; diluted 1:500); and anti-glutamate decarboxylase (GAD) 65 (AB5082, rabbit polyclonal, Millipore, Billerica, MA; diluted 1:1000 and ab203063, rabbit polyclonal, Abcam, Cambridgde, UK; diluted 1:500).Primary antibodies were diluted in washing buffer containing 3% normal goat serum or normal horse serum. After incubation with the primary antibody, sections were then rinsed in buffer and incubated for 2 h at room temperature with biotinylated horse anti-mouse immunoglobulin G (BA2000, Vector laboratories, Burlingame, CA; diluted 1:250 in washing buffer), biotinylated goat anti-guinea pig immunoglobulin G (BA7000, Vector laboratories, Burlingame, CA; diluted 1:250 in washing buffer) or biotinylated goat anti-rabbit immunoglobulin G (BA1000, Vector laboratories, Burlingame, CA; diluted 1:250 in washing buffer). After several washes in buffer, sections were incubated for 1 h at room temperature with avidin–biotin peroxidase complex (ImmunoPure ABC, Pierce, Rockford, IL; diluted 1:125). Peroxidase activity was revealed with 0.01% hydrogen peroxide, using 3,3ʹ-diaminobenzidine (Sigma, St Louis. MO; 0.05%). Finally, sections were mounted, dehydrated and counterstained with methyl green (Sigma, St. Louis, MO) and coverslipped with DEPEX (VWR, Rannor, Pennsylvania). The slides were observed with a digital microscope (Zeiss). Immunostaining was absent when the primary antibody was omitted. All the experimental groups were assayed in parallel.

For immunofluorescence, free-floating sections were incubated overnight at 4 °C with one of the following primary antibodies at the dilutions described above: anti-NeuN; anti-parvalbumin; anti-vGLUT1; anti-vGLUT2; and anti-vGAT, in washing buffer containing 3% normal goat serum. Sections were then rinsed in buffer and incubated for 2 h at room temperature with 3% normal goat serum and the following secondary antibodies: Alexa Fluor 594-conjugated goat anti-mouse (A11005, Molecular Probes, Madrid, Spain), Alexa Fluor 594-conjugated goat anti-rabbit (A11012, Molecular Probes, Madrid, Spain) and Alexa Fluor-647-conjugated goat anti-guinea pig (A21450, Molecular Probes, Madrid, Spain). The nuclei were counterstained with DAPI (4′,6-diamidino-2-phenylindole) (Sigma, San Louis; MO; diluted 1:80). Sections were mounted on histological slides and coverslipped with ProLong^®^ Gold antifade reagent (Life technologies, Carlsbad, CA).

Possible alterations in the structure and distribution of the extracellular matrix constituents of perineuronal nets, which surround PV-positive GABAergic interneurons, were assessed by double fluorescence staining using fluorescein*-Wisteria floribunda lectin* (WFL) (Vector Laboratories, 1:500) that recognizes N-acetylgalactosamine residues in the extracellular matrix. This staining was used in combination with immunocytochemistry using anti-GAD-65 antibodies or anti-SMI-32 antibodies. GAD-65 antibodies label subpopulations of inhibitory neurons, whereas anti-SMI-32 antibodies label a subset of excitatory (a subpopulation of pyramidal neurons) and inhibitory neurons (a subpopulation of interneurons). The sections were incubated overnight at 4 °C with primary antibodies and WFL at the dilutions described previously and were then incubated with the following secondary antibodies: Alexa Fluor 594-conjugated goat anti-mouse (A11005, Molecular Probes, Madrid, Spain) or Alexa Fluor 594-conjugated goat anti-rabbit (A11012, Molecular Probes, Madrid, Spain). The sections were stained with DAPI, after rinsing in PB, and were then treated with Autofluorescence Eliminator Reagent (Chemicon) to reduce autofluorescence according to the manufacturer’s instructions, mounted in antifade mounting medium (ProlongGold, Invitrogen) and studied by confocal microscopy (Zeiss, 710).

Fluorescence images were acquired using a LSM710 confocal laser-scanning microscope (Carl Zeiss MicroImaging, Jena, Germany). Z sections were recorded at 1-μm intervals through separate channels, and subsequently, ZEN 2012 software (Zeiss) was used to construct composite images from each optical series by combining the images recorded through the different channels. Finally, sections adjacent to those used for histochemistry and immunohistochemistry were stained with thionine or cresyl violet to reveal the borders between the different layers and hippocampal fields and to examine possible changes in the general cytoarchitectural characteristics under the different experimental conditions.

### Metabolomics: tissue processing and reagents, sample treatments, analysis methods, data processing, statistical analysis and compound identification

For metabolomics studies, we followed the same protocol for the removal of the brains as that described above for the analysis of immersion-fixed tissue. However, after removal of the brains (*n* = 15), they were kept on ice, while the left hippocampi were dissected. The samples were then frozen on liquid nitrogen and stored at −80 °C until processed. Tissue samples were divided into three groups (*n* = 5 per group) according to the PT that had elapsed when brains were extracted from the animals: 30 min, 2 h and 5 h.

Reverse-osmosed ultrapure water, used to prepare all the aqueous solutions, was obtained “in-house” from a Milli-Qplus185 system (Millipore, Billerica, MA, USA). LC–MS grade methanol and analytical grade formic acid were from Sigma-Aldrich (Steinheim, Germany). Analytical grade ammonia hydroxide (30% ammonium in high purity water) was acquired from Panreac Quimica SA (Barcelona, Spain). Reagents for derivatization (*O*-methoxyamine hydrochloride and BSTFA:TMCS, 99:1 (Sylon BFT)) were purchased from Sigma-Aldrich (Steinheim, Germany) and Supelco (Bellefonte, PA, USA), respectively. Standard mix for GC–MS, containing grain fatty acid methyl esters (C8:0-C22:1, n9), and analytical grade heptane were purchased from Fluka Analytical (Sigma-AldrichChemie GmbH, Steinheim, Germany). Silylation-grade pyridine was from VWR International BHD Prolabo (Madrid, Spain). Methyl-tert-butyl-ether (MTBE) used for hydrophobic compounds extraction was acquired from Sigma-Aldrich (Steinheim, Germany).

#### Sample treatment procedure for metabolite extractions

Brain samples were prepared for LC–MS and GC–MS at CEMBIO (Madrid, Spain). The method used for the extraction was previously developed and validated at CEMBIO for lung tissue (Naz et al. 2013) and was selected, after testing, from several different extraction conditions because it also provided a higher number of reproducible signals for brain. Briefly, approximately 30 mg of hippocampus tissue was used for the study, and samples were stored at −80 °C until the analysis. The solvent used for the tissue homogenization was MetOH: H_2_O 50% (v/v), (1:10 tissue/solvent). Samples were homogenized using a TissueLyser LT homogenizer (Qiagen, Germany). For extraction, 100 μL of hippocampus tissue homogenate was vortex-mixed with 320 μL of methanol for 2 min after which 80 μL of MTBE was added. Vials were immediately capped and placed on a shaker for 1 h at room temperature. The extracted samples were then centrifuged at 4000*g* at 20 °C for 20 min, and 90 μL of supernatant was transferred to a chromatography vial for LC–MS analysis and 300 μL to a separate vial for GC–MS analysis. For LC–MS analysis, the supernatant was injected directly into the system.

For GC–MS, 300 μL of supernatant was evaporated to dryness using a SpeedVac Concentrator System. Methoxymation was performed with *O*-methoxyamine hydrochloride (15 mg/mL in pyridine) and vigorously vortex-mixed for 5 min. For silylation, 20 μL of BSTFA:TMCS (99:1) was added, vortex-mixed for 5 min, and capped vials were placed in the oven at 70 °C for 1 h. Prior to injection, 100 μL of heptane containing C18:0 methyl ester (10 ppm) as IS was added.

Quality control samples (QC) were prepared by pooling equal volumes (approx. 100 μL) of hippocampus homogenate from each of the 15 samples. Five QC samples were independently prepared in parallel by dividing up the total volume of this pooled QC. These QCs were treated as the rest of the samples and were analyzed throughout the run to provide a measurement not only of the stability and performance of the system, but also of the reproducibility of the sample treatment procedure.

#### Hippocampus fingerprinting by LC-QTOF-MS

Metabolic fingerprinting performed in LC-QTOF-MS was carried out with a liquid chromatography (LC) system (1200 series, Agilent Technologies, Waldbronn, Germany) consisting of a degasser, two binary pumps, thermostated autosampler, maintained at 15 °C and a column oven. Based on previously optimized conditions for lipidomic analysis (Whiley et al. 2012), 10 μL of extracted hippocampus samples was injected into a reverse phase column (Agilent; Poroshell EC-C8, 15 cm × 2.1 mm, 2.7 μm) with a guard column (Supelco Ascentis Express C8, 0.5 cm × 2.1 mm, 2.7 μm) thermostated at 60 °C. The gradient used for the analysis consisted of a mobile phase A (5 mM ammonium formate in Milli-Q water) and mobile phase B (5 mM ammonium formate in methanol/isopropanol, 85:15) pumped at 0.5 mL/min. Initial conditions at time 0 were 82% B, increasing to 96% B in 30 min. This was then maintained until 38 min. The gradient then increased to 100% B by 38.5 min and was maintained for 2 min until 40.5 min. The starting condition was returned to by 42 min, followed by an 8-min re-equilibration time, taking the total run time to 50 min. Data were collected in positive ESI mode on a QTOF (6520 Agilent Technologies) with a scan rate of 1.02 scans/s operated in full scan mode from 100 to 1200 *m*/*z*. The capillary voltage was 3500 V, the nebulizer gas flow rate was 10.0 L/min, the source temperature was 350 °C, and its pressure was 40 psi. Two reference masses were used in the course of the whole analysis: *m*/*z* 121.0509 (protonated purine, C_5_H_4_N_4_) and *m*/*z* 922.0098 (HP-921, C_18_H_18_O_6_N_3_P_3_F_24_) for positive ionization mode. Masses were continuously infused into the system to provide constant mass correction. Samples were analyzed randomly in the run.

#### Hippocampus fingerprinting by GC-Q-MS

The analysis of the derivatized extracts was performed with an Agilent GC instrument (7890 A) coupled to an inert mass spectrometer with Triple-Axis Detector (5975 C, Agilent Technologies). The injection volume of derivatized samples was set at 2 μL using an Agilent autosampler (7693). Samples were automatically injected in split mode, with a split ratio 1:10, into an Agilent deactivated glass wool split liner. Separation of the compounds was achieved using a 10 m J&W precolumn (Agilent Technologies) integrated with a 122 − 5332G column: DB5-MS 30 m length, 0.25 mm i.d. and 0.25 μm film consisting of 95% dimethyl/5% diphenyl polysiloxane (Agilent Technologies). The Helium carrier gas flow was held constant at 1 mL/min. The lock of the retention time (RTL) relative to the internal standard (methyl stearate C18) peak at 19.66 min was performed. The initial column oven temperature was set at 60 °C (maintained for 1 min), then raised by 10 °C/min until it reached 325 °C and was held at this temperature for 10 min before cooling down. The injector and the transfer line temperatures were set at 250 and 280 °C, respectively. MS system: The electron impact ionization operating parameters were set as follows: filament source temperature, 230 °C; electron ionization energy, 70 eV. Mass spectra were collected from 50 to 600 m/z at a scan rate of 2 spectra/s. Data were acquired using the Agilent MSD ChemStation software (Agilent Technologies).

#### Data processing

For a global profiling, raw data collected by GC–MS and LC–MS were reprocessed using different software packages.

##### GC–MS data treatment

Total ion chromatograms (TICs) were inspected based on the quality of the chromatograms and internal standard signal. First, samples were processed with MassHunter Workstation GC/MS Translator software version B.04.01, in order to reformat data files as necessary to conform to the MassHunter Quantitative data analysis format. Deconvolution and metabolite identification of raw data collected by GC/MS analysis were performed by Agilent MassHunter Unknowns Analysis Tool 7.0. A chemical identity was assigned to the compounds by the software by searching in two target libraries: Fiehn library version 2008 and the CEMBIO in-house spectral library by comparing both retention time (RT) and spectra extracted during the deconvolution against each compound included in the library (Kind et al. 2009). In addition, a commercial spectral library—NIST (National Institute of Standards and Technology) library 2.2 version 2014—was used for comparing non-identified compounds. Those with spectrum score >80% and concordant retention index (n-alkane scale) were putatively identified according to NIST. Data obtained by the Unknown Analysis Tool were aligned using MassProfiler Professional B.12.1 (Agilent Technologies). Assignment of the target ions and signal integration was performed with Agilent MassHunter Quantitative. Before any statistical calculation, sample concentrations were normalized by IS abundance in order to minimize the response variability introduced by the instrument. Moreover, data were filtered by coefficient of signal variation (CV) in QCs, considering values lower than 30% as acceptable.

##### LC–MS data treatment

The resulting data file obtained by the LC–MS was cleaned of background noise and unrelated ions by the Molecular Feature Extraction (MFE) tool in MassHunter Profinder software. The MFE then creates a list of possible components that represent the full TOF mass spectral data features, which are the sum of co-eluting ions that are related by charge-state envelope, isotopic distribution and/or the presence of different adducts and dimmers. Several parameters of the algorithm were set for data extraction, applying 300 counts as limits for the background noise. Moreover, the algorithm was applied to find co-eluting adducts for the same possible compound, selecting +H, +Na, +NH_4_ and neutral water loss as possible adducts for positive ionization. Then, MFE aligns features across all sample files using mass and retention time (RT) to build a unison spectrum for each compound group, enabling the next step of the analysis, the re-extraction of the batch files—also known as Batch Recursive Feature Extraction (RFE). RFE performs MFE again and then uses the mass and RT of the results to improve the quality of the target list referred to as Find by Ion (FbI) and the quality of the final compound group list. Thereafter, the metabolomic data obtained were filtered by CV in QCs, keeping metabolites with CVs in QCs lower than 30%.

### Statistical analysis

Differences among the groups were investigated using univariate data analysis (UVDA) and multivariate data analysis (MVDA). For UVDA, differences between the three post-mortem stages were evaluated for each individual metabolite using MATLAB (R2015a, MathWorks) by one-way ANOVA (*p* ≤ 0.05) validated by Levene’s test, testing the homogeneity of variance and trusting only *p* values with *p* Levene higher than 0.05. Where statistical differences appeared by using ANOVA for any of the comparisons, differences among the means were compared using Tukey’s test in order to find which comparisons were statistically significant and the trend of the metabolite concentration. For multiple comparison correction, the Benjamini–Hochberg method was applied to all *p* values to control the false-positive rate at level *α* = 0.05. Metabolites whose levels had a tendency to change according to the PT interval were maintained even if they did not pass this restrictive correction for enhanced information. The F statistic was obtained to reflect the PT effects. Finally, a post hoc pairwise comparison with two-sample *t* test was performed to conclude whether the metabolite is significant or not in a comparison (Krzywinski and Altman 2014). All these parameters are represented in the graphics in Supplementary Fig. S1 of the Supplementary Information. For MVDA, SIMCA-P + 12.0 (Umetrics, Umea, Sweden) was used. As a first exploratory step, unsupervised principal component analysis (PCA) was generated for a preliminary evaluation of natural clustering of the samples and examination of grouping of QC samples that is indicative of stability in the analysis performance. In addition, supervised models such as partial least square discriminant analysis (PLS-DA) and orthogonal PLS discriminant analysis (OPLS-DA) were also built. The quality of the models was assessed by the explained variance (R^2^) and the predicted variance (Q^2^), supplied by the software. Finally, statistically significant variables were selected according to the variable importance in projection (VIP) and jackknifing confidence interval from the OPLS-DA models. The metabolites that were assumed to be the most meaningful ones for the comparison were those with a VIP score ≥ 1 and a jackknife not including 0. Finally, fold change was calculated for each metabolite, in order to estimate the variation in the abundance of the metabolites within each comparison. In the present study, the percentage of change showed a general positive trend, which means that the level of metabolites increased over time. Metabolites with a percentage of change greater than 30% were considered statistically significant.

### Compound identification

Putative identification of statistically significant features for the LC–MS analysis was achieved by searching the list of *m*/z against the databases available online, such as METLIN (http://metlin.scripps.edu), lipidsMAPS (http://lipidMAPS.org) and KEGG (http://www.genome.jp/kegg/), all of which have been integrated into an in-house developed search engine, CEU MassMediator (http://ceumass.eps.uspceu.es/mediator). HMDB (http://hmdb.ca) was also accessed for supplementary information. Features that were putatively assigned to lipids from the databases were based on: (1) mass accuracy (maximum error mass 20 ppm); (2) isotopic pattern distribution; (3) possibility of cation and anion formation; and (4) adducts formation (Godzien et al. 2013).

## Results

### Neuroanatomical and neurochemical studies

Histological studies were performed in mouse brain harvested at different post-mortem times (PT, 0 h, 0 min), 30 min, 1 h, 2 h, 3 h and 5 h and subsequently fixed by immersion in 4% paraformaldehyde in PB versus mouse brain transcardially perfused with the same fixative. We have evaluated the effects that the post-mortem delay in the fixation procedure might have on the distribution and intensity of the staining after different histological procedures. Since small changes visualized using histological, histochemical and immunohistochemical techniques are typically found from experiment to experiment, even using sections from the same brains, subtle changes are difficult to interpret. Thus, we were only looking for large, obvious changes. Therefore, we have qualitatively analyzed the staining patterns in the different subdivisions of the dorsal hippocampus (dentate gyrus and CA1, CA2 and CA3) from Bregma −1.46 to −2.30 (Paxinos and Franklin 2001).

#### Neuronal markers

##### General markers

**Fig. 1:**
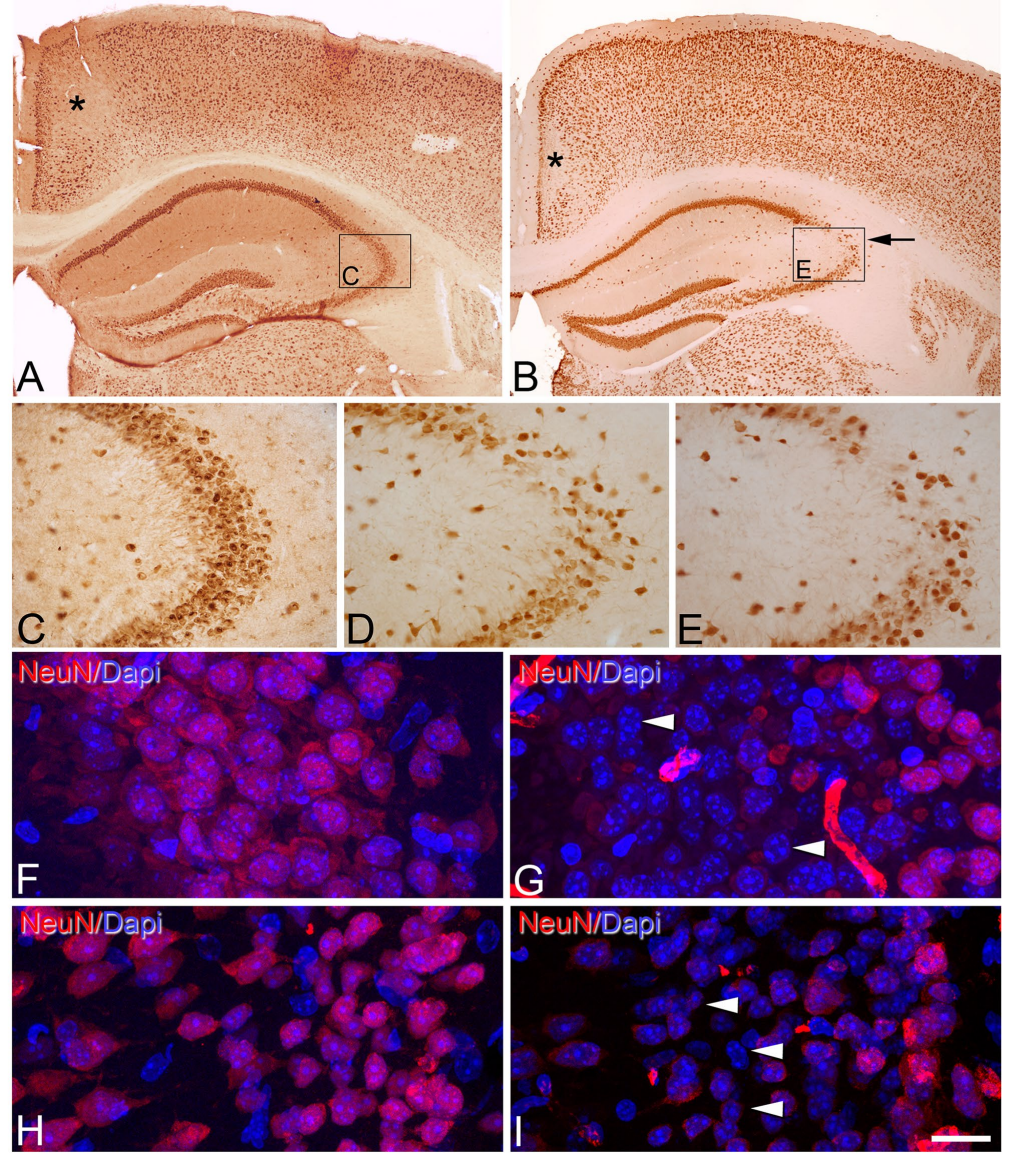
*Post-mortem* time-related alterations of NeuN immunostaining in mouse cerebral cortex. **a, b** Low-magnification photomicrographs showing NeuN immunostaining of sections from the neocortex and hippocampus of the brain of mice fixed by immersion immediately after death (PT 0 h, 0 min) (**a**) or by immersion after 5 h PT (**b**). Note that in **b** there are zones with a reduction of NeuN immunostaining in the retrosplenial cortex (*asterisk*) and the CA3 hippocampal region (*arrow*). However, in the retrosplenial cortex, these changes were already observed at 0 h (0 min), whereas in CA3, it was observed from 30 min to 5 h. **c, d, e** High-magnification photomicrographs showing the reduction in the number of NeuN-ir neurons in the *stratum pyramidale* of CA3 in animals with 30 min PT (**d**) and, more markedly, with 5 h PT (**e**) versus immersion-fixed brain tissue that was extracted immediately after death (PT 0 h, 0 min) (**c**) or perfusion-fixed tissue (see Supplementary Fig. 1S2 A, B). *Rectangles* in **a** and **b** indicate the areas of magnification in **c** and **e**, respectively. **f–i** Confocal images taken from the pyramidal cell layer of CA3 (**f, g**) and layers II and III of retrosplenial cortex (**h, i**) of sections immunostained for NeuN and counterstained with DAPI in perfusion-fixed tissue (**f, h**) and immersion-fixed tissue 5 h PT (**g, i**). *Arrow heads* point to DAPI staining of the nucleus of neurons devoid of NeuN immunostaining. *Scale bar* (in **i**): 330 µm in (**a**), B; 80 µm in (**c–e**); 15 µm in (**f–i**)

We first used an antibody for NeuN, which specifically recognizes a soluble, nuclear, neural vertebrate DNA-binding protein that is present in the vast majority of mature neurons in both the central and peripheral nervous systems of several vertebrate species, including humans (Mullen et al. 1992; Wolf et al. 1996; Sarnat et al. 1998). Immunostaining for NeuN in the immersion-fixed brain material labeled virtually all neurons, which allowed the cytoarchitectonic features of the different regions and the limits between them to be distinguished. Although no quantitative evaluation of the neuronal number or intensity of immunostaining was performed, we found differences in the pattern of immunostaining for NeuN in the hippocampus between brains fixed by immersion (Fig. 1a–c), and between these brains and perfused brains (Supplementary Fig. S2). As shown in Fig. 1, there was a clear decrease in NeuN immunostaining in the *stratum pyramidale* of CA3 close to the boundary with CA2 in animals from 30 min PT (Fig. 1d) onward that was more pronounced at 5 h PT (Fig. 1e). However, in the brain of mice fixed by immersion immediately after death (PT 0 h, 0 min), NeuN immunostaining was similar to that found in perfused animals (see Supplementary Fig. S2, A, B).

##### Markers for subpopulations of neurons

We also examined whether the fixation procedure and PT affected the staining of various neuronal subpopulations using immunocytochemistry with SMI-32, parvalbumin (PV) and calbindin-D28K (CB) antibodies, and the histochemical staining for NADPH-d.

SMI-32 is a marker of neuronal subtypes including pyramidal cells and interneurons in the neocortex and hippocampus (Mikuni et al. 1998). In line with these studies, in our material from perfused animals, SMI-32-ir neuronal somata were mainly found in the subgranular zone and polymorphic layer of the dentate gyrus (Supplementary Fig. S3) and in the *strata pyramidale and oriens* of the CA fields. In the brain of animals fixed by immersion after different *post-mortem* periods, a similar pattern of SMI-32 immunostaining of cell somata and processes to that of perfused brains was found (Supplementary Fig. S3).

The distribution patterns and apparent density of the different subpopulations of neurons positive for PV, CB and NADPH-d found in the hippocampus were in agreement with previous studies (Celio 1990; Celio and Heizmann 1981; Tamamaki et al. 2003; Maskey et al. 2012; DeFelipe 1997). However, as shown in Supplementary Figs. S4, S5 and S6, there were marked changes including increases and decreases in the labeling of elements (cell bodies, neuronal processes or puncta) depending on the hippocampal region and layer examined in immersion-fixed tissue from 30 min to 5 h PT as compared to tissue from perfused animals. These changes occurred after 30 min in the case of CB-immunostaining and NADPH-d histochemical staining and after 2 h in the case of PV. For example, among other changes, there was a clear reduction in the labeling of PV-immunostaining processes and an increase in the NADPH-d staining in the stratum pyramidale of CA1 and CA3, after 2 h in the case of PV and after 30 min in the case of NADPH-d histochemical staining.

##### Labeling of glutamatergic axon terminals

**Fig. 2:**
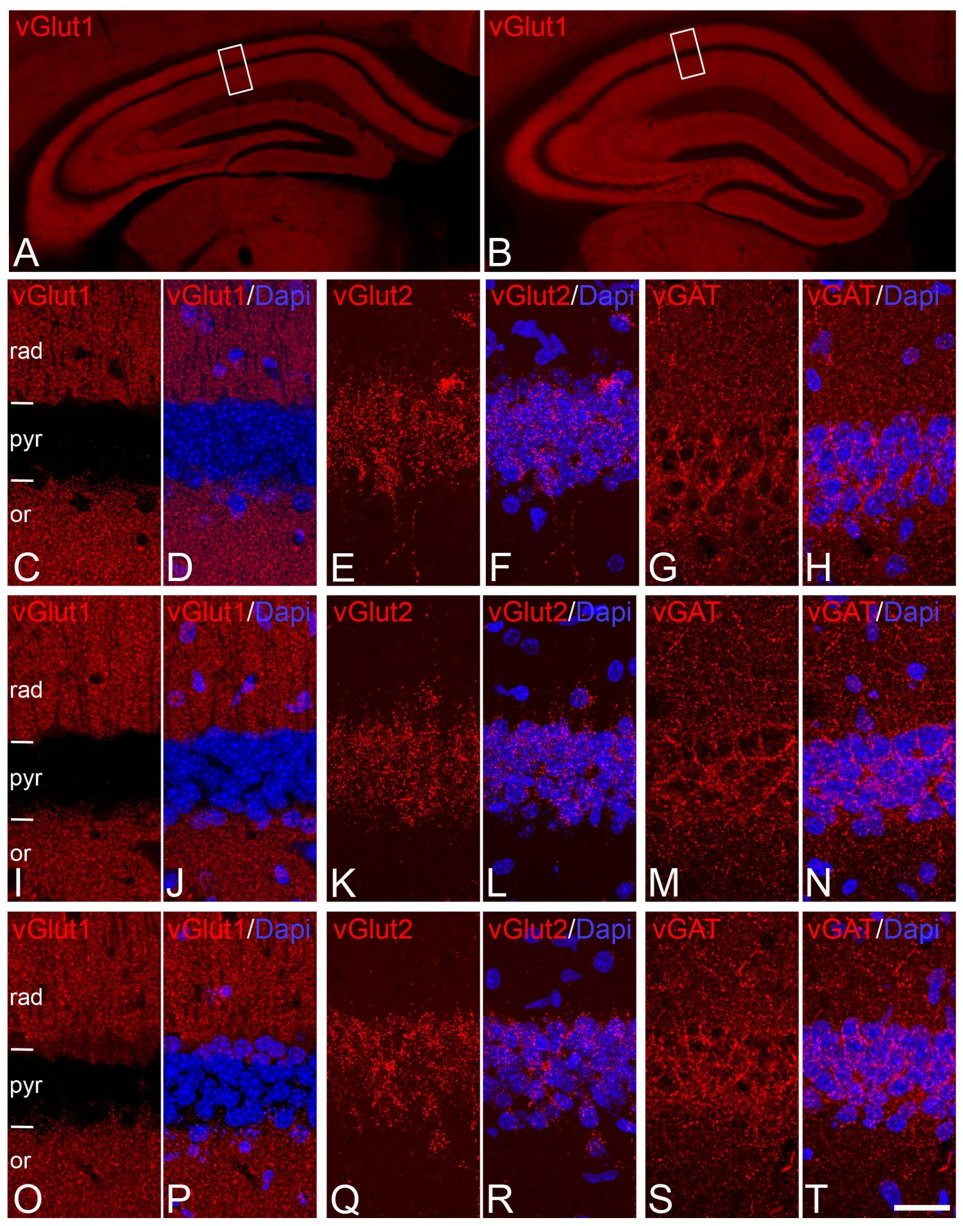
Distribution of glutamatergic axon terminals and GABAergic axon terminal immunoreactivity in the CA1 hippocampal region. **a, b** Low-magnification photomicrographs showing the similar distribution and intensity of immunostaining of vGLUT1 in the hippocampus of perfusion-fixed animals (**a**) and brains fixed by immersion after 5 h PT (**b**). **c**–**t** Pairs of confocal images from the CA1 region taken from sections immunostained for vGLUT1 (C,D,I,J,O,P), v-GLUT2 (E,F,F,L,Q,R), vGAT (G,H,M,N,S,T) and counterstained with DAPI from perfusion-fixed brains (**c**–**h**) or brains fixed by immersion after 30 min (**i**–**n**) or 5 h (**o**–**t**) PT. *Rectangles* in **a** and **b** indicate the areas of magnification in **c** and **o**, respectively. Note that vGlut1 and vGlut2 terminals distribute in a roughly complementary way in CA1. Also note the apparent absence of changes in the distribution of vGlut1, vGlut2 and vGAT between the brains fixed with different procedures and PTs. or, stratum oriens; pyr, stratum pyramidale; rad, stratum radiatum. *Scale bar* (in **t**): 400 µm in (**a, b**); 17 µm in (**c**–**t**)

In perfused brain material, immunostaining for vesicular glutamate transporters vGlut1 and vGlut2—the two major isoforms in the brain (Nakamura et al. 2005)—labeled numerous punctate elements in the neuropil of neocortex and hippocampus that, according to previous studies, are known to correspond to excitatory terminals that form asymmetric synapses (Fagg and Foster 1983; Fonnum 1984; Danbolt 2001; Ni et al. 1995; Hisano et al. 2000; Fujiyama et al. 2001; Herzog et al. 2001; Kaneko and Fujiyama 2002; Varoqui et al. 2002; Kaneko et al. 2002; Miyazaki et al. 2003; Hioki et al. 2003; Fremeau et al. 2001, 2004; Takamori 2006). According to these studies, vGlut1 and vGlut2 were found partially segregated showing a roughly complementary distribution. vGlut1-ir punctate elements were widely distributed throughout the *stratum lacunosum moleculare, radiatum* and *oriens* of the hippocampus, as well as the molecular layer of the DG and the polymorphic layer of hilus (Fig. 2). By contrast, vGlut2-ir terminals were chiefly concentrated in *stratum pyramidale* (Fig. 2) and the molecular layer of the dentate gyrus. Although no quantification of either the terminals or intensity of immunostaining was performed, these patterns of vGlut1 and vGlut2 immunostaining were similar between immersion-fixed tissue with different PT and tissue from perfused animals (Fig. 2).

##### Labeling of GABAergic axon terminals

**Fig. 3:**
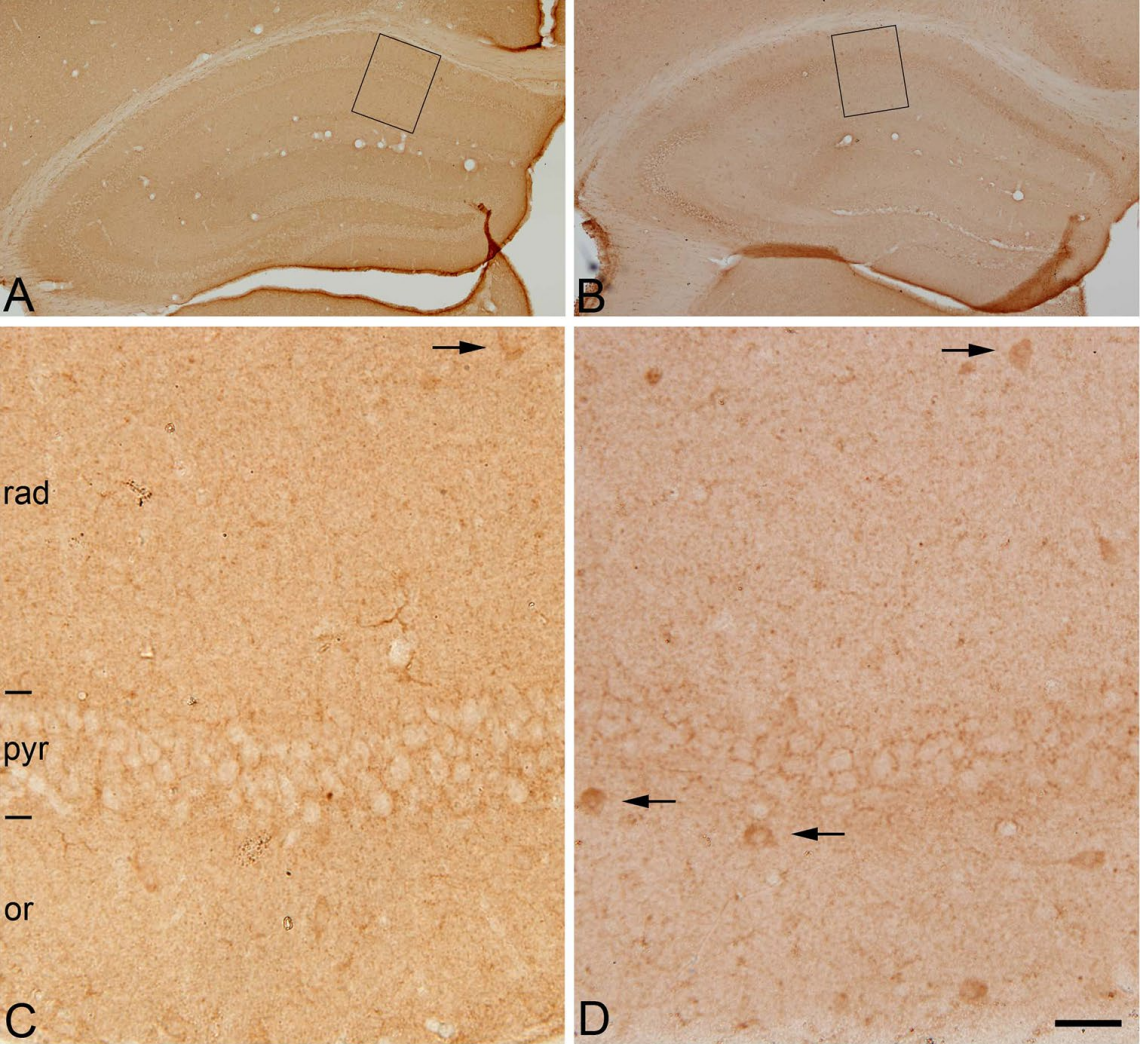
Post-mortem time-related alterations of GAD-65-immunostaining in mouse hippocampus. Low (**a, b**)- and higher (**c, d**)-magnification photomicrographs (*rectangles* in **a** and **b**, respectively) showing the distribution patterns of GAD-65-immunoreactivity of sections through the hippocampus of mice brains fixed by perfusion (**a, c**) or by immersion after 5 h PT (**b, d**). Note that in perfusion-fixed tissue (**c**) GAD-65 immunoreactivity is present mainly in punctate structures (presumptive axon terminals) distributed in the neuropil or surrounding cell bodies of unlabeled neurons. Occasionally, some cell bodies are also immunostained (*arrows*). In post-mortem brain tissue, the pattern of immunostaining is similar to that found in perfused brain tissue with the exception that a larger number of GAD-65-ir neurons are stained (**d**) (*arrows*). or, stratum oriens; pyr, stratum pyramidale; rad, stratum radiatum. *Scale bar*: 280 µm in (**a, b**); 30 µm in (**c, d**)

Immunostaining for the vesicular GABA transporter (vGAT), known to correspond to GABAergic inhibitory axon terminals (Chaudhry et al. 1998; Minelli et al. 2003), labeled numerous terminal-like puncta in the neuropil in the cerebral cortex. In the hippocampus, vGAT-ir puncta were particularly evident in the dentate gyrus at the border between the granule cell layer and the molecular layer as well as in the *stratum pyramidale* of the CA hippocampal regions. These patterns of vGAT immunostaining were again similar between immersion-fixed tissue and tissue from perfused animals (Fig. 2). Furthermore, we used immunohistochemistry for the 65-kDa isoform of the GABA-synthesizing enzyme glutamic acid decarboxylase (GAD-65), which is particularly prominent in axon terminals, whereas no or very few labeled cell bodies are immunostained (Esclapez et al. 1994). This pattern of GAD-65 immunostaining was observed in tissue from perfused animals (Fig. 3a, c), whereas in immersion-fixed tissue from 30 min to 5 h, numerous labeled cell bodies were observed in the hippocampus (Fig. 3b, d) as well as in many other brain regions (not shown). No apparent changes were seen at 0′ h, 0′ min.

##### Labeling of extracellular matrix

We have also examined whether the fixation procedure and the PT delay affected components of the extracellular matrix using labeling with *Wisteria floribunda lectin* (WFL) that recognizes N-acetylgalactosamine residues in the extracellular matrix in perineuronal nets that wrap PV-positive GABAergic interneurons (Foster et al. 2014; Kosaka and Heizmann 1989) both in neocortex and in hippocampus. We found that WFA labeling of perineuronal net-like structures is well preserved in immersion-fixed tissue with the different PT and similar to that in brains from perfused animals (Supplementary Figs. S3 and S6). This suggests that the integrity of the extracellular matrix surrounding PV-positive GABAergic neurons is not significantly altered during the first 5 h PT.

#### Glial markers

**Fig. 4:**
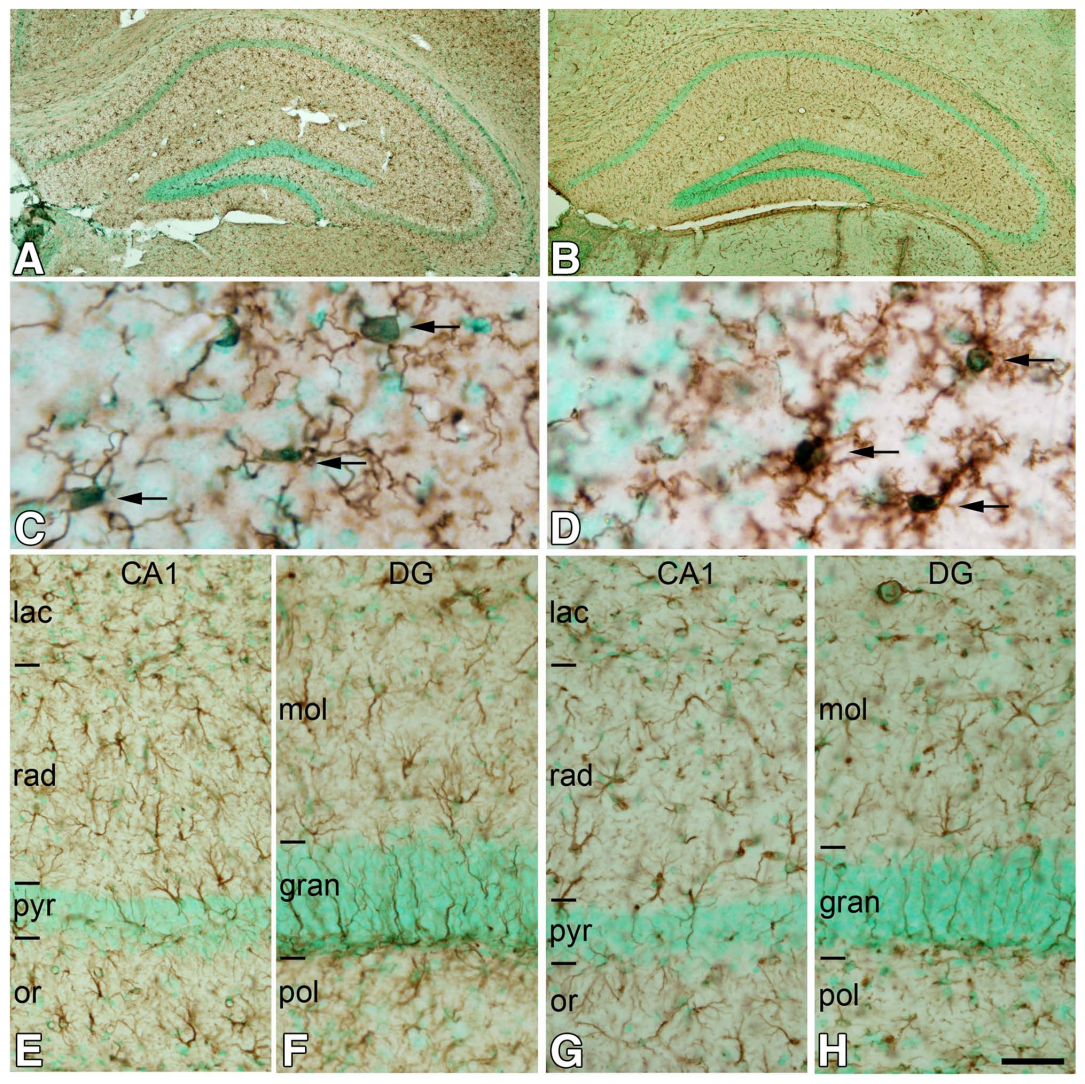
Post-mortem time-related changes in microglial and astroglial cells in the hippocampus. **a, b** Low-magnification photomicrographs showing Iba-1 (microglial cells; **a**) and GFAP immunoreactivity (astrocytes; **b**) in immersion-fixed tissue 5 h PT counterstained with methyl *green*. **c**, **d** High-magnification photomicrographs showing that Iba-1-ir microglial cells have larger cell bodies (*arrows*) and thick, retracted processes in immersion-fixed tissue 5 h PT (**d**) as compared to perfusion-fixed tissue (**c**). **e**–**h** Low-magnification photomicrographs of CA1 (**e, g**) and the dentate gyrus (**f, h**) showing the absence of apparent differences in the patterns of GFAP immunostaining in the brain of animals fixed by immersion after 5 h PT (**g, h**) compared to animals fixed by perfusion (**e, f**). DG, dentate gyrus; gran, granular layer; lac, stratum lacunosum moleculare; mol, molecular layer; or, stratum oriens; pol, polymorphic layer; pyr, stratum pyramidale; rad, stratum radiatum. *Scale bar* (in **h**): 300 µm in (**a, b**); 16 µm in (**c**, **d**); 40 µm in (**e**–**h**)

Finally, we examined whether the fixation procedure and the PT delay affected astrocytes and microglial cells in sections, respectively, immunostained for glial fibrilar acid protein (GFAP) and the EF-hand protein Iba1, which is specifically expressed in microglia (Imai et al. 1996). No general differences in the labeling of these two populations of glial cells were found in the most neocortical and hippocampal regions between the perfusion-fixed tissue and tissue fixed by immersion after different PT (Fig. 4). However, morphological differences in Iba1-ir microglial cells were found between perfusion-fixed tissue and tissue that was immersion-fixed from 2 to 5 h in those regions in which a decrease in the neuronal expression of NeuN was noted, including the retrosplenial cortex and the CA3 hippocampal field (Fig. 4c, d). Here, in line with the morphological descriptions of microglial cells in different pathological conditions (Soltys et al. 2001; Kabadi et al. 2012) (Zhan et al. 2008), in the present study Iba1-ir cells showed a proinflammatory phenotype ranging from hypertrophic microglia (with larger cell bodies and thick, retracted processes) to bushy microglia (with numerous but short and poorly ramified processes of different diameters forming thick bundles around their enlarged cell bodies) (Fig. 4d).

In summary, the marked differences that we observed in the tissue fixed by immersion after different PTs when compared with the perfusion-fixed tissue were: (1) an obvious focal decrease in the immunostaining for NeuN in the stratum pyramidale of CA3, particularly close to the boundary with CA2 after 30 min; (2) marked changes in PV- and CB-immunostaining, and NADPH-d histochemical staining including increases and decreases in the labeling of elements depending on the hippocampal region and layer examined, after 30 min in the case of CB-immunostaining and NADPH-d histochemical staining and after 2 h in the case of PV; (3) an increase in the number of cell bodies immunostained for GAD-65 throughout the hippocampus after 30 min; and (4) morphological changes in Iba1-ir microglial cells (proinflammatory phenotype) after 2 h in those regions in which a focal decrease in the neuronal expression of NeuN was observed. All these differences were more apparent in the 5-h PT tissue.

### Metabolomics studies

Regarding metabolomics studies, an initial PCA plot of both LC–MS and GC–MS from metabolomic data was generated to check the clustering of the QC samples and determine the robustness of the methodology. The QC samples were tightly clustered for both analyses, indicating the stability and reproducibility of the system.

Seven hundred and fifty-six and 58 entities were obtained in LC–MS and GC–MS, respectively. Data sets after filtering consisted of 704 entities in LC–MS and 45 identified compounds in GC–MS. PCA modeling showed a tendency of sample grouping according to time stage. Additionally, supervised PLS-DA models were built for the three groups and demonstrated a clear separation between them, suggesting that metabolite levels change with time after death. PCA plots and PLS-DA plots for GC–MS and LC–MS are illustrated in supplementary Fig. S7. For OPLS-DA models, differences between post-mortem stages were evaluated by pairs and validated by cross-validation, leaving one sample out per group. Results are represented in Fig. S8, including the R^2^ and Q^2^ for each comparison model.

Parallel to multivariate analysis, univariate statistical analysis was performed in order to determine the statistical significance of each metabolite separately. After univariate and multivariate data analysis, 22 and 14 compounds were shown to be statistically significant for LC–MS and GC–MS, respectively. Finally, identified metabolites are listed in Table 1 arranged in different biochemical categories, reflecting features such as retention time, the monoisotopic mass, molecular formula, error mass, among others. In the table, a general positive trend can be observed, which means that the level of metabolites increased over time. Compounds identified by LC–MS presented a bigger increase between 30 min and 2 h PT since the increment showed at 5 h PT was nearly as high as in the first period of time.

**Table 1:**
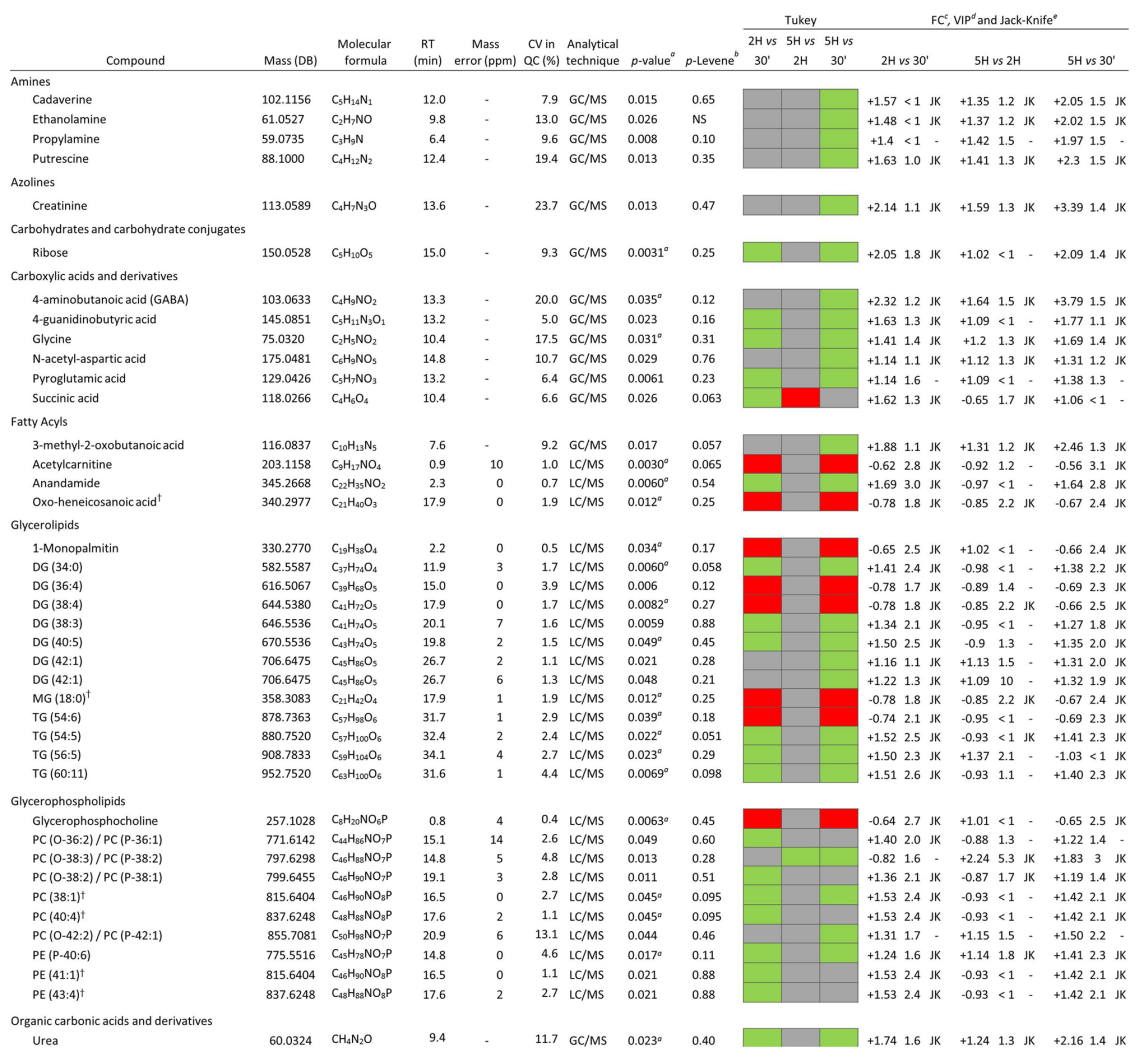
Metabolites that showed statistical significance any of the post-mortem comparisons at different times in hippocampus

^†^Alternative identification. Besides all of the metabolic changes observed in this study, it is important to take into account all the compounds found in the analysis which showed no significant alterations over time. All these metabolites are summarized in Table 2, next to a summary of all the metabolites found to be statistically significant

^a^CV, coefficient of variation of the metabolites in the QC samples

^b^ *p* value, corrected *p* value by Benjamini–Hochberg test correction

^c^ *p* Levene, *p* Levene was considered as significant at values higher than 0.05 in order to test homogeneity of variances, *NS* nonsignificant

^d^Tukey’s test results colored by significance of the metabolite in the specific comparison (green means the metabolite is significant and is up-regulated, red means the metabolite is significant and is down-regulated, and gray means the metabolite is not significant)

^e^FC, fold change in the specified comparison; the sign indicates the direction of change

^f^VIP, VIP values higher than 1 were considered as significant

^g^Jackknife confidence interval

**Table 2:** Metabolite classification according to their behavior within time

Changes	No changes
Amines	Amino acids, peptides and analogues
*Cadaverine*	*Alanine*
*Ethanolamine*	*Glutamic acid*
* Propylamine*	*Glutamine*
*Putrescine*	*Isoleucine*
Amino acids, peptides and analogues	* Leucine*
* GABA*	*Serine*
* 4-guanidinobutyric acid*	* Threonine*
* Glycine*	Cholesterol and cholesterol esters
* N-acetyl-aspartic acid*	Fatty acyls
* Pyroglutamic acid*	*Arachidonic acid*
Azolines	*Myristic acid*
* Creatinine*	* Oleic acid*
Carbohydrates and carbohydrate conjugates	* Palmitic acid*
*Ribose*	* Stearic acid*
Dicarboxylic acids and derivatives	Organic acids
* Succinic acid*	*3-hydroxybutyric acid*
Fatty acyls	* Fumaric acid*
*3-methyl-2-oxobutanoic acid*	*Glycolic acid*
*Acetylcarnitine*	*Malic acid*
* Anandamide*	*Phosphoric acid*
Glycerolipids	Phospholipids and lysophospholipids
Glycerophospholipids	Polyols
Organic carbonic acids and derivatives	* Myo-inositol*
	Sphingolipids
	Vitamins
	Nicotinamide

## Discussion

Autopsy tissue is rarely obtained within 2 h of post-mortem. In the present study, we considered 30 min to be a feasible optimal lower limit to obtain post-mortem human brain tissue, taking into account the time required to follow necessary procedures between death and dissection. To evaluate the effects of these post-mortem periods, we compared mouse brain tissue fixed after different PT periods of up to 5 h. According to the neuroanatomical and metabolomic results obtained in this study, significant metabolomic changes already began at 2 h PT, whereas neuroanatomical observations revealed that although the general patterns of labeling for the markers used here were preserved, there were selective changes produced in immersion-fixed tissue depending on the post-mortem time. These changes occurred mostly at 5 h PT. Since some neurochemical characteristics of brain tissue that was extracted immediately after death and fixed by immersion were different compared with the perfused brains, these results indicate that the method of fixation per se (i.e., perfused versus non-perfused brains) may influence the results of the immunostaining.

**Fig. 5:**
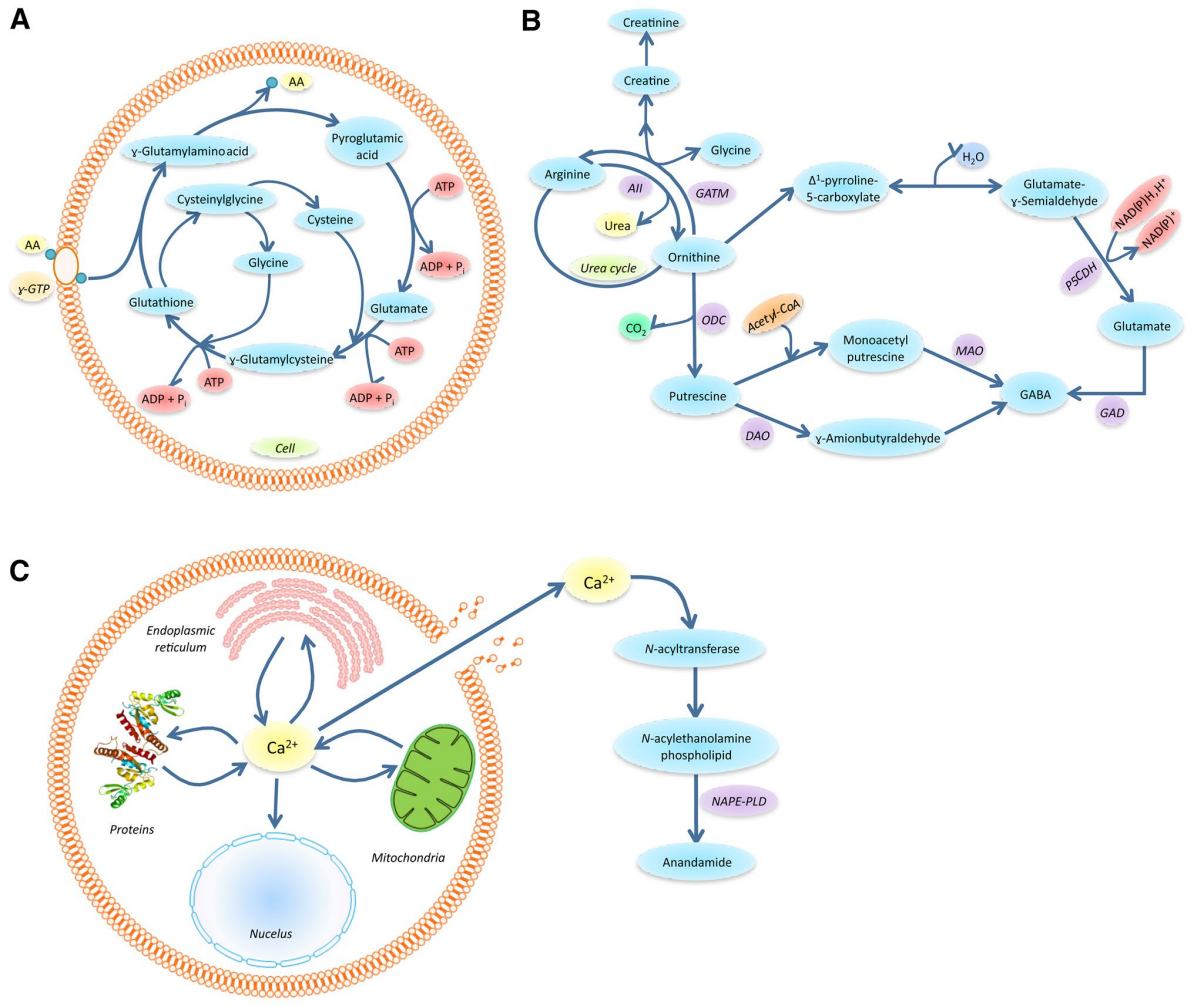
**a** *γ*-glutamyl cycle for transporting amino acids coupled to the degradation of glutathione. AA is the amino acid being transported inside the cell by the *ɣ-*glutamyl transpeptidase, *ɣ-GTP*. **b** Reactions involved in creatine/creatinine metabolism and urea cycle coupled to catabolism of L-arginine and L-ornithine. The enzymes involved in the different steps are: *AII* arginase II, *GATM* L-arginine:glycine amidinotransferase, *ODC* ornithine decarboxylase, *OAT* ornithine, *DAO* diamine oxidase, *MAO* monoamino oxidase, *GAD* glutamic acid decarboxylase, pyrroline-5-carboxylate (*PC5*) dehydrogenase. **c** Activation of anandamide synthesis by calcium release. The enzyme involved is a specific phospholipase D (*NAPE-PLD)*

The levels of many relevant metabolites evaluated by metabolic procedures increased until 2 h PT and then their levels either remained stable up to 5 h PT (as was the case for pyroglutamic acid, anandamide and urea) or decreased (as was the case for glycerophosphocholine). Other metabolites—such as GABA, creatinine, *N*-acetyl-aspartic acid, putrescine and cadaverine—reached higher levels at 5 h PT. Cholesterol and cholesterol esters and other compounds like arachidonoylglycerol were stable. Therefore, the present results indicate that caution is required when performing neuroanatomical and metabolomics post-mortem quantitative studies, especially in anatomical areas with a high sensitivity for oxidative damage and conditions of anoxia. Although assessing whether there were any correlations between anatomical and metabolomics results was not the aim of this study, what follows is a discussion of the possible significance of the changes found in our study as a whole (referring to the present study unless otherwise specified). The increased concentrations of some amino acids observed post-mortem by GC–MS analysis may occur due to catabolic reactions of glutathione (GSH). The amino acids coming from the tripeptide GSH are glycine, cysteine and pyroglutamic acid (Dringen 2000). There was a clear trend for these amino acids to increase in concentration, with the exception of cysteine, which is made unstable by the oxidation of the thiol group and is thus not detected. One of the main physiological roles of GSH is the cellular defense against reactive oxygen species (ROS). These radicals are generated at high rates within the brain, which, compared with other organs, seems to be especially endangered by the generation and detoxification of ROS (Dringen 2000; Dringen et al. 2000). The hippocampus is especially sensitive to oxidative stress; it has a high oxygen consumption and is rich in lipids with unsaturated fatty acids, the targets of lipid peroxidation (Dringen et al. 2000). Furthermore, the hippocampus has a relatively low content of antioxidants (Evans 1993). Therefore, GSH antioxidant activity is an essential task for brain function (Dringen 2000). GSH is predominantly metabolized through the γ-glutamyl cycle (Fig. 5a), which acts by regulating the transit of amino acids through the blood–brain barrier, safeguarding brain homeostasis (Hawkins et al. 2006). Resynthesizing one mole of GSH requires the hydrolysis of three moles of ATP (Harish et al. 2011). As a consequence, the lack of ATP related to the post-mortem hypoxia state can compromise the glutathione system. Various papers state that brain glutathione content drops rapidly after death, reflecting the fact that its metabolism is influenced by post-mortem interval (Donaldson and Lamont 2015; Perry et al. 1981; Harish et al. 2011; Epstein et al. 2013). According to the explanation above, increased glycine and pyroglutamic acid levels in the hippocampus tissues are consistent with GSH hydrolysis. These findings can affect the interpretation of previous studies about altered glutathione metabolism in age-related neurodegenerative diseases (Pearce et al. 1997) (Aoyama and Nakaki 2013).

In our tissue, we have not found apparent differences in the general distribution of different populations of neurons identified by PV-, CB-immunostaining or NADPH-d histochemical staining, between the perfusion-fixed tissue and tissue fixed by immersion after up to 5 h of PT delay. However, there were marked changes in PV- and CB-immunostaining, and NADPH-d histochemical staining in immersion-fixed tissue, after 30 min in the case of CB-immunostaining and NADPH-d histochemical staining and after 2 h in the case of PV, as compared to tissue from perfused animals. These changes were particularly evident at 5 h PT and included increases and decreases in the staining of labeled cell bodies, neuronal processes or puncta depending on the hippocampal region and cortical layer examined.

In a previous study carried out in rhesus macaque monkeys with longer PT periods (up to 48 h), it was found that PV-ir cells presented a low definition of immunostaining of the fine dendritic processes (Lavenex et al. 2009). In addition, the authors reported that SMI-32 immunoreactivity was also greatly reduced in somas and dendrites of neurons located in several zones of hippocampus (Lavenex et al. 2009), whereas we did not find any significant change in our immersion-fixed material up to 5 h PT.

The PT delay also affected NeuN immunostaining in the CA3 hippocampal region; it was particularly reduced after 5 h of PT. In this region, DAPI staining revealed that the loss of NeuN immunostaining occurred in the absence of apparent cell death. This is in agreement with previous reports describing a depletion of NeuN immunoreactivity in hypoxic conditions in sudden infant deaths (Lavezzi et al. 2013) and in mouse brains after cerebral ischemia (Unal-Cevik et al. 2004). There seems to be a regional vulnerability to pathological conditions based on the high rate of oxidative activity and relatively low antioxidant capacity (Igarashi et al. 2001). The CA1 and CA3 hippocampal fields and dentate gyrus are especially sensitive to oxidative damage (Chang et al. 2012; Uysal et al. 2012). For example, peripheral oxidative stress has been strongly associated with diminished hippocampal volume—a finding that has been most reliably demonstrated in the CA3 and DG (Lindqvist et al. 2014). In a PT study on AD, it was suggested that an oxidative stress pathway starts in the CA3 subfield and then progresses to other hippocampal regions and the neocortex (Cruz-Sanchez et al. 2010).

In regions with an obvious decrease in NeuN immunostaining, we found that microglial cells showed a proinflammatory phenotype after different PT and more markedly at 5 h PT. In conditions of lack of ATP related to post-mortem hypoxia state, it is possible that the microglial cells are activated and this status was more evident at 5 h PT. We found an increase in GABA and biological compounds from the metabolism of arginine such as urea, polyamines, creatine and glutamate at 5 h PT. These metabolites can play an important role in the innate immune response, through the activation or anti-inflammatory response of microglial cells (Pocock and Kettenmann 2007; Colton 2009; Ahn et al. 2011; Fontainhas et al. 2011). Microglial cells arginase I, an inducible cytoplasmic form, and arginase II, a constitutively mitochondrial form (Yu et al. 2003; Colton et al. 2006), and the microglial expression of Arg1 and iNOS can vary with M1 and M2 microglial phenotypes. Arg1 and iNOS can be up-regulated during the progression of amyotrophic lateral sclerosis, and in motor neurons, Arg1 may confer protection from disease processes (Lewis et al. 2014). The neuroprotective M2 state has been related to an anti-inflammatory alternative activation phenotype, which participates in repair and resolution to tissue homeostasis in pathological conditions (Colton 2009).

It was noted that the compounds synthesized from amino acids increased considerably over time, while only small changes in the levels of precursor of amino acids were observed. 3-methyl-2-oxobutanoic acid comes from valine, leucine and isoleucine pathways. Its percentage change was 146% in the comparison 5 h *versus* 30 min, but no evidence of its precursors was found, probably because they degraded rapidly.

Creatinine is a compound that is worthy of attention since its percentage of change was 239%. Creatinine is formed by spontaneous and unidirectional non-enzymatic conversion from creatine and, above all, from creatine phosphate, which serves as energy storage for the body. By transporting phosphates between sites of ATP production and ATP consumption, the creatine/phosphoryl-creatine/creatinine system acts as an intracellular energy shuttle and helps to maintain the energy balance in all tissues with high-energy demands, like brain (Donaldson and Lamont 2015). The level of free creatine in the body is low because it is rapidly converted to phosphoryl-creatine by enzyme creatine kinase, as well as in the second step of the process leading to the final product creatinine (Gallant et al. 2006). To obtain creatine phosphate, the hydrolysis of two ATP molecules into AMP is required. As a consequence, when the levels of ATP drop, creatine phosphate rapidly degrades to creatinine. Since no system is activated to remove creatinine after death, this substance turns out to be a clear marker that there is no more anabolic activity, and there is catabolism alone without any form of excretion. For this reason, creatinine provides evidence of the time elapsed after death and it is used as an index in forensic analysis. Since creatine/creatinine metabolism has recently been linked to neurodegenerative diseases (Wyss and Kaddurah-Daouk 2000), PT should be considered in experimental designs.

In addition, the considerable increase of creatinine highlighted in this study could be interpreted as being a consequence of the increased number of metabolites involved in the urea cycle, from which creatine derives—in particular, a clear increase in glycine as previously explained. The connection between creatinine and urea pathways is shown in Fig. 5b. To date, little is known about post-mortem changes in the level of urea, but such accumulation of urea might be due to continued ammonia formation after death, as a consequence of protein and amino acid breakdown by either enzyme or bacterial activity (Jenkins 1953). Since brain is a very well-isolated system, shortly after death, the enzymatic activity prevails over bacterial activity and putrefaction is not accentuated.

The multiple metabolic fates of arginine make it one of the most versatile amino acids. Indeed, arginine is metabolized through a complex and highly regulated set of pathways that leads to the formation of many biologically important compounds: urea, polyamines, agmatine, creatine and thus creatinine, and glutamate, which is a precursor for the synthesis of GABA (gamma-aminobutyric acid) (Morris 2007). The chemical derivatization of arginine is difficult due to the low reactivity of the guanidinium group. For this reason, it is rarely identified in GC analysis, but most of its metabolites were found in the present investigation. Researchers have recently become aware that protein turnover can release various arginine derivatives (Morris 2006). This consideration, together with the increase of arginine levels due to post-mortem protein breakdown, may account for the increasing rate of production of urea, polyamines, creatine and glutamate, providing explanation for our results. The pathways interconnected by arginine are shown in Fig. 5b (Caron et al. 1987).

A further point to note is that putrescine and cadaverine belong to the family of biogenic polyamines, which are present in relatively high levels in the brain, where they play an essential role (Burkard et al. 1963). Measuring polyamines in post-mortem brain tissue is worthwhile considering recent evidence implicating polyamines in neurological disorders (Chen et al. 2009). Previous studies suggest that polyamine levels are stable in rodent autopsied brain up to 24 h PT and no correlation between polyamine levels and PT interval was observed (Morrison et al. 1995). By contrast, we found a pronounced increase in putrescine and cadaverine, with a high percentage of change (130 and 105%, respectively) over time. The synthesis of putrescine involves an initial input from ornithine decarboxylase (ODC), and its substrate, ornithine, is generated from arginine (Morris 2007). As a result, putrescine can be considered to be one of the metabolites whose high concentration can be related to the increase in arginine level resulting from the protein breakdown that takes hold after death. Moreover, according to published evidence (Seiler 2000), ischemic and hypoxic conditions associated with death cause an increased activity of ODC, accompanied by an increase of putrescine concentration in brain. Interestingly, it is not only the activity of ODC that increases; arginine decarboxylase activity increases too. Regarding cadaverine, there is an endogenous source of this diamine in the mouse and it is actually synthesized in mouse brain (Stepitak and Dolezalo 1974). In addition, both putrescine and cadaverine are substrates of various amine oxidases (Schmidt-Glenewinkel et al. 1977). Several studies have been carried out to elucidate the diamine oxidase (DAO)-mediated metabolism of polyamines. It was demonstrated that, after death, polyamine metabolism operated by DAO gradually slows down. This is compatible with the appreciable increase in post-mortem polyamines levels in mouse brain (Stepita-Klauco and Dolezalova 1974). Moreover, it is interesting to note that putrescine also contributes to formation of GABA by means of diamine oxidase.

Inside GABAergic neurons, GABA is generated from glutamic acid in a single enzymatic step catalyzed by glutamate decarboxylase (GAD). Rate limiting this step occurs via steric hindrance associated with ATP. This could explain the increase in GABA post-mortem when ATP levels drop (Karam 2012). It should also be noted that, since the conversion from glutamate to GABA is anaerobic, it can continue after death. Circumstantial evidence for the role of GABA in anoxic conditions comes from studies demonstrating that the lack of oxygen induces a considerable increase in the GABA content in brain (Nilsson 1993). Taking this into account, the positive trend of GABA was predictable. Other investigations based on the measurement of post-mortem changes in mouse brain reported that GABA is one of the amino acids that rapidly increase in mouse brain after death (Perry et al. 1981). This is in line with the obvious increase in the labeling of cell bodies with immunocytochemistry using anti-GAD-65 antibodies that we observed throughout the hippocampus in post-mortem brain tissue.

There was a trend to increase one derivative of aspartic acid, N-acetylaspartic (NAA), after death (Simmons et al. 1991). NAA is the second most concentrated molecule in the brain after glutamate, and like glutamate, it may function as a neurotransmitter (Yan et al. 2003). NAA, which is synthesized primarily in neurons, is the second most concentrated molecule in the brain after glutamate, and like glutamate, it may function as a neurotransmitter (Yan et al. 2003). However, its role in these cells remains unclear. One of the main hypotheses is that NAA is involved in cell signaling together with NAAG, controlling the interactions of brain cells and preserving the nervous system (Baslow 2000). NAA is also considered to be an important marker of neuronal viability in many cerebral pathologies, where a decline in its concentration is interpreted as a sign of neuronal or axonal dysfunction or death (Tyson and Sutherland 1998). Nevertheless, an increment in NAA concentration also induces many alterations such as oxidative stress, Canavan disease or genotoxicity and protein interaction due to an increase in nitric oxide produced by the elevated concentration of NAA (Surendran and Bhatnagar 2011). Compared with other organs, the brain has a high content of lipids, some two-thirds of which are phospholipids (Ohkubo and Tanaka 2010). Hippocampal pyramidal neurons represent by far the most abundant type of neuron in the hippocampus, and their dendritic arbor is covered by dendritic spines. Dendritic spines are the main target of excitatory glutamatergic synapses and are considered to be critical for cognition, learning and memory. Thus, many researchers are interested in the study of possible alterations of dendritic spines in brain diseases (DeFelipe 2015). These structures contain a complex mixture of ions, lipids, proteins and other signaling molecules which must be continually restored (Sorra and Harris 2000). In fact, it is well known that cholesterol and sphingolipids (SL) are enriched in dendritic spines. The extraction of cholesterol or inhibition of its synthesis leads to the disappearance of dendritic spines, which proves that cholesterol is a core component of dendritic spines (Dotti et al. 2014). Cholesterol and cholesterol esters were identified by two techniques (GC–MS and LC–MS), but differences over time were not statistically significant after data analysis, indicating that degradation 5 h PT is negligible. This is in line with the study by Williams et al. (1978); they found no appreciable changes in the density and morphology of spines in the dendritic arbors of pyramidal neurons of the mouse with fixation latencies of 5 min to 6 h, but, with latencies of more than 6 h, they observed a reduction in the density of dendritic spines and morphological changes of these structures. SL make up approximately 20% of the hippocampus, including sphingomyelin, cerebrosides, cerebroside sulfates and gangliosides. SL act as important signaling molecules in neuronal tissue, and they have received wide attention due to the relatively high levels of gangliosides found. However, recent studies have shown that simple SL, such as ceramide, sphingosine-1-phosphate and glucosylceramide (GlcCer), also play important roles in neuronal function such as in regulation of neuronal growth rates, differentiation and cell death (Buccoliero and Futerman 2003). Ceramide, a second messenger in neurons, contributes to spine plasticity thanks to its capacity to favor membrane fusogenicity promoting receptor clustering (Kronke 1999). Although ceramide is the best characterized SL, its glucosyl derivative, GlcCer also has important functions since it regulates the rate of axonal and dendritic growth (Boldin and Futerman 1997; Harel and Futerman 1993). However, GlcCer is found in low concentrations since it is a metabolic intermediate in the biosynthetic pathway leading to formation of other GSLs. The proper formation and long-term maintenance of neuronal connectivity are crucial for correct functioning of the brain. The long-lasting stability of dendrite and spine structure in the nervous system is highly dependent on the actin cytoskeleton, which is particularly well developed in these structures (Koleske 2013). Sphingomyelin, one of the most abundant SL in neuronal membranes, has an important role in the spine membrane–cytoskeleton cross talk since it modulates membrane binding and the activity of main regulators of the actin cytoskeleton at synapses (Dotti et al. 2014). Sphingosine 1-phosphate is a bioactive lipid that controls a wide range of the cellular processes described above, and it is also involved in cytoskeletal organization. Moreover, it plays a pivotal role is the formation of memory (Kanno et al. 2010). However, no significant differences in any of these compounds were observed over time.

Phospholipids, such as phosphatidylcholine (PC) and phosphatidylethanolamine (PE), are the two major phospholipid classes of brain membranes, and from previous experimental studies, reduction of total PC and PE mass might be expected during brain degeneration post-mortem. Nevertheless, due to the high content of these phospholipids, only extensive breakdown (>10%) of these compounds will be visible as significant changes in tissue homogenates (Klein 2000). Numerous studies have addressed the question of whether the hypothesized activations of phospholipases are visible in post-mortem tissue. The enzyme phospholipase A_2_ (PLA_2_) recognizes the *sn-*2 fatty acyl bond of phospholipids and catalyzes the hydrolysis of the bond, releasing free fatty acids and lysophospholipids (Dennis 1994). Since no Lyso-PC was statistically significant, we can assume that phospholipase A_2_ (PLA_2_) does not change its activity post-mortem.

As described in the literature, triacylglycerol (TAG) is quantitatively a minor component of the whole brain, representing <0.2% of total brain lipid, but its levels can become increased in pathological conditions. However, low levels of TAG in brain do not indicate low metabolic activity for this neutral lipid fraction: Low concentration of free fatty acids (FA) in total brain lipid composition could be due to the direct exchange of acyl chains between TAG, not releasing free FA in this process; thus, the lack of a statistically significant outcome for FA could be explained (Cook 1981; Smith and White 1968). Given that TAG is present in low concentrations in brain tissue, it can be assumed that all the diacylglycerols (DAG) and monoacylglycerols (MAG) reflected as statistically significant come from glycerophospholipids. Brain tissue contains several classes of glycerophospholipids, with different rates of turnover depending on their structure and localization. Glycerophospholipids are vulnerable to being catabolized by phospholipases A_1_, A_2_, C and D yielding different second messengers. Phospholipase C (PLC) performs the hydrolysis of the phosphodiester bond at the *sn*-3 position leading to the formation of DAG (Farooqui et al. 2000). Since DAGs are products of phospholipase C, a global increase in its activity can be assumed. The two statistically significant monoglycerides obtained, monopalmitin [MG(16:0)] and monostearin [MG(18:0)], were previously described in brain tissue (Rowe 1969).

Glycerophosphocholine (GPCh) is present in relatively high concentration in brain tissue. It was previously reported that GPCh concentration decreased progressively in the first hour PT in the striatum and cortex of rat brain (Jope and Jenden 1979). As can be observed in Table 1, the abundance of GPCh decreased until 2 h PT, and after 5 h, its abundance was no lower than at 2 h.

The endogenous cannabinoid system has been studied in great detail in the hippocampal formation, where there is a high density of cannabinoid binding sites (Hajos et al. 2000). The endocannabinoid (eCB) system is a retrograde signaling system composed of lipid mediators, the endocannabinoids and cannabinoid receptors type 1 (CB1) and type 2 (CB2). The main expression sites of these receptors are different: CB1 is expressed in brain, peripheral nervous system, gastrointestinal tract, heart, liver, adipose tissue, lungs, adrenal glands, smooth and skeletal muscle, male and female reproductive systems, bone and skin; CB2 is expressed in a very low concentration in brain, restricted to specific neuronal cells, and is present in high levels in microglia and astrocytes and abundantly expressed in immune system cells (monocytes, macrophages, and B and T cells) and peripheral organs (spleen, tonsils, thymus gland, mast cells, keratinocytes and gastrointestinal system). Finally, the eCB system also consists of enzymes involved in the synthesis and degradation of endocannabinoids. The main endocannabinoids of the CB1 receptor are 2-arachidonoylglycerol (2-AG) and anandamide (AEA) (Piyanoya et al. 2015). The concentration of these compounds in the hippocampus is approximately twice that found in the cortex (Maccarrone et al. 2002). AEA and 2-AG belong to the lipid families of *N*-acylethanolamines (NAEs) and 2-monoacylglycerols (2-MAGs), respectively. The phospholipid precursors of NAEs, *N-*acylethanolamine phospholipids (NAPE), are accumulated in the rat brain rapidly post-mortem (Natarajan et al. 1986), which led to the hypothesis that accumulation of these substances may be due to neuronal death. This concept is consistent with previous findings, based on several animal models (Schmid et al. 1995). The synthesis of these compounds is calcium-regulated, and as described above, after death, the calcium contained in cells is released by cellular lysis. When calcium concentrations rise, the main enzymes for generation of AEA and 2-AG are stimulated, that is, N-acyltransferase and phosphoinositide-specific phospholipase C, respectively. Their activation results in the formation of a variety of NAPE and DAG species, respectively, and the corresponding NAE and 2-MAG species are released by the activation of a NAPE-specific phospholipase D and a stereoselective DAG lipase (Hansen et al. 2001). All these reactions are represented in Fig. 5c. However, only AEA levels increased until 2 h PT, and then, its levels were stable, while 2-AG levels did not change with time. These results are in agreement with a previous study by Palkovits et al. (2008), in which the concentration of AEA in human hippocampal tissue with 2–3 h PT was found to be almost double that of 1–1.5 h PT, and no further increase was observed at 4–6 h PT (Palkovits et al. 2008).

In conclusion, the present results indicate that PT delays of up to 5 h do not affect the general pattern of labeling of a number of neuronal systems and glial cells in the hippocampus, and this is also the case for many metabolites. However, there are also highly significant changes—not only in the levels of a variety of metabolites, but also PT-dependent changes in the immunostaining or histochemical staining using certain markers of neurons and microglia that are commonly used to analyze the human brain both in health and in disease. Thus, knowledge of which metabolites are stable, and which are susceptible to change, as well as information regarding the neuroanatomical/neurochemical characteristics of neurons and glia at any given PT delay, is essential in order to make interpretations of studies using human brain autopsies.

## Electronic supplementary material

Below is the link to the electronic supplementary material.

Supplementary material 1 (DOCX 5613 KB)

## References

[CR1] Ahn SK, Hong S, Park YM, Lee WT, Park KA, Lee JE (2011). Effects of agmatine on hypoxic microglia and activity of nitric oxide synthase. Brain Res.

[CR2] Alonso-Nanclares L, Gonzalez-Soriano J, Rodriguez JR, DeFelipe J (2008). Gender differences in human cortical synaptic density. Proc Natl Acad Sci USA.

[CR3] Aoyama K, Nakaki T (2013). Impaired Glutathione Synthesis in Neurodegeneration. Int J Mole Sci.

[CR4] Baslow MH (2000). Functions of *N*-acetyl-l-aspartate and *N*-acetyl-l-aspartylglutamate in the vertebrate brain: role in glial cell-specific signaling. J Neurochem.

[CR5] Blazquez-Llorca L, Garcia-Marin V, DeFelipe J (2010). GABAergic complex basket formations in the human neocortex. J Comp Neurol.

[CR6] Blazquez-Llorca L, Merchan-Perez A, Rodriguez J-R, Gascon J, DeFelipe J (2013). FIB/SEM technology and Alzheimer’s disease: three-dimensional analysis of human cortical synapses. J Alzheimers Dis.

[CR7] Boldin S, Futerman AH (1997). Glucosylceramide synthesis is required for basic fibroblast growth factor and laminin to stimulate axonal growth. J Neurochem.

[CR8] Buccoliero R, Futerman AH (2003). The roles of ceramide and complex sphingolipids in neuronal cell function. Pharmacol Res.

[CR9] Buell SJ (1982). Golgi-Cox and rapid golgi methods as applied to autopsied human brain tissue: widely disparate results. J Neuropathol Exp Neurol.

[CR10] Burkard WP, Gey KF, Pletscher A (1963). Diamine oxidase in brain of vertebrates. J Neurochem.

[CR11] Caron PC, Kremzner LT, Cote LJ (1987). GABA and its relationship to putrescine metabolism in the rat-brain and pancreas. Neurochem Int.

[CR12] Celio MR (1990) Calbindin D-28k and parvalbumin in the rat nervous system. Neurosci 35(2):375–475 10.1016/0306-4522(90)90091-h 2199841

[CR13] Celio MR, Heizmann CW (1981). Calcium-binding protein parvalbumin as a neuronal marker. Nature.

[CR14] Chang B-J, Jang B-J, Son TG, Cho I-H, Quan F-S, Choe N-H, Nahm S-S, Lee J-H (2012). Ascorbic acid ameliorates oxidative damage induced by maternal low-level lead exposure in the hippocampus of rat pups during gestation and lactation. Food Chem Toxicol.

[CR15] Chaudhry FA, Reimer RJ, Bellocchio EE, Danbolt NC, Osen KK, Edwards RH, Storm-Mathisen J (1998). The vesicular GABA transporter, VGAT, localizes to synaptic vesicles in sets of glycinergic as well as GABAergic neurons. J Neurosci.

[CR16] Chen GG, Turecki G, Mamer OA (2009). A quantitative GCMS method for three major polyamines in postmortem brain cortex. J Mass Spectrom.

[CR17] Colton CA (2009). Heterogeneity of Microglial Activation in the Innate Immune Response in the Brain. J Neuroimmune Pharmacol.

[CR18] Colton CA, Mott RT, Sharpe H, Xu Q, Van Nostrand WE, Vitek MP (2006). Expression profiles for macrophage alternative activation genes in AD and in mouse models of AD. J Neuroinflamm.

[CR19] Cook HW (1981). Metabolism of triacylglycerol in developing rat-brain. Neurochem Res.

[CR20] Cruz-Sanchez FF, Girones X, Ortega A, Alameda F, Lafuente JV (2010). Oxidative stress in Alzheimer’s disease hippocampus: A topographical study. J Neurol Sci.

[CR21] Danbolt NC (2001). Glutamate uptake. Prog Neurobiol.

[CR22] DeFelipe J (1997). Types of neurons, synaptic connections and chemical characteristics of cells immunoreactive for calbindin-D28K, parvalbumin and calretinin in the neocortex. J Chem Neuroanat.

[CR23] DeFelipe J (2015). The dendritic spine story: an intriguing process of discovery. Front Neuroanat.

[CR24] Del Rio MR, DeFelipe J (1994). A study of SMI 32-stained pyramidal cells, parvalbumin-immunoreactive chandelier cells, and presumptive thalamocortical axons in the human temporal neocortex. J Comp Neurol.

[CR25] Dennis EA (1994). Diversity of group types, regulation, and function of phospholipase A2. J Biol Chem.

[CR26] Donaldson AE, Lamont IL (2015). Metabolomics of post-mortem blood: identifying potential markers of post-mortem interval. Metabolomics.

[CR27] Dotti GC, Esteban JA, Ledesma MD (2014). Lipid dynamics at dendritic spines. Front Neuroanat.

[CR28] Dringen R (2000). Metabolism and functions of glutathione in brain. Prog Neurobiol.

[CR29] Dringen R, Gutterer JM, Hirrlinger J (2000). Glutathione metabolism in brain—Metabolic interaction between astrocytes and neurons in the defense against reactive oxygen species. Eur J Biochem.

[CR30] Durrenberger PF, Fernando S, Kashefi SN, Ferrer I, Hauw JJ, Seilhean D, Smith C, Walker R, Al-Sarraj S, Troakes C, Palkovits M, Kasztner M, Huitinga I, Arzberger T, Dexter DT, Kretzschmar H, Reynolds R (2010). Effects of antemortem and postmortem variables on human brain mRNA quality: a BrainNet Europe study. J Neuropathol Exp Neurol.

[CR31] Epstein AA, Narayanasamy P, Dash PK, High R, Bathena SPR, Gorantla S, Poluektova LY, Alnouti Y, Gendelman HE, Boska MD (2013). Combinatorial assessments of brain tissue metabolomics and histopathology in rodent models of human immunodeficiency virus infection. J Neuroimmune Pharmacol.

[CR32] Esclapez M, Tillakaratne NJ, Kaufman DL, Tobin AJ, Houser CR (1994). Comparative localization of two forms of glutamic acid decarboxylase and their mRNAs in rat brain supports the concept of functional differences between the forms. J Neurosci.

[CR33] Fagg GE, Foster AC (1983). Amino-acid neurotransmitters and their pathways in the mammalian central nervous-system. Neurosci.

[CR34] Farooqui AA, Horrocks LA, Farooqui T (2000). Glycerophospholipids in brain: their metabolism, incorporation into membranes, functions, and involvement in neurological disorders. Chem Phys Lipids.

[CR35] Fonnum F (1984). Glutamate—a neurotransmitter in mammalian brain. J Neurochem.

[CR36] Fontainhas AM, Wang M, Liang KJ, Chen S, Mettu P, Damani M, Fariss RN, Li W, Wong WT (2011) Microglial Morphology and Dynamic Behavior Is Regulated by Ionotropic Glutamatergic and GABAergic Neurotransmission. Plos One 6(1). doi:10.1371/journal.pone.0015973 10.1371/journal.pone.0015973 PMC3026789 21283568

[CR37] Foster NL, Mellott JG, Schofield BR (2014). Perineuronal nets and GABAergic cells in the inferior colliculus of guinea pigs. Front Neuroanat.

[CR38] Fremeau RT, Troyer MD, Pahner I, Nygaard GO, Tran CH, Reimer RJ, Bellocchio EE, Fortin D, Storm-Mathisen J, Edwards RH (2001). The expression of vesicular glutamate transporters defines two classes of excitatory synapse. Neuron.

[CR39] Fremeau RT, Voglmaier S, Seal RP, Edwards RH (2004). VGLUTs define subsets of excitatory neurons and suggest novel roles for glutamate. Trends Neurosci.

[CR40] Fujiyama F, Furuta T, Kaneko T (2001). Immunocytochemical localization of candidates for vesicular glutamate transporters in the rat cerebral cortex. J Comp Neurol.

[CR41] Gallant M, Rak M, Szeghalmi A, Del Bigio MR, Westaway D, Yang J, Julian R, Gough KM (2006). Focally elevated creatine detected in amyloid precursor protein (APP) transgenic mice and Alzheimer disease brain tissue. J Biol Chem.

[CR42] Godzien J, Ciborowski M, Whiley L, Legido-Quigley C, Ruperez FJ, Barbas C (2013). In-vial dual extraction liquid chromatography coupled to mass spectrometry applied to streptozotocin-treated diabetic rats. Tips and pitfalls of the method. J Chromatogr A.

[CR43] Hajos N, Katona I, Naiem SS, Mackie K, Ledent C, Mody I, Freund TF (2000). Cannabinoids inhibit hippocampal GABAergic transmission and network oscillations. Eur J Neurosci.

[CR44] Hansen HH, Schmid PC, Bittigau P, Lastres-Becker I, Berrendero F, Manzanares J, Ikonomidou C, Schmid HH, Fernaández-Ruiz JJ, Hansen HS (2001). Anandamide, but not 2-arachidonoylglycerol, accumulates during *in vivo* neurodegeneration. J Neurochem.

[CR45] Harel R, Futerman AH (1993). Inhibition of sphingolipid synthesis affects axonal outgrowth in cultured hippocampal-neurons. J Biol Chem.

[CR46] Harish G, Venkateshappa C, Mahadevan A, Pruthi N, Bharath MMS, Shankar SK (2011). Glutathione metabolism is modulated by postmortem interval, gender difference and agonal state in postmortem human brains. Neurochem Int.

[CR47] Hawkins RA, O’Kane RL, Simpson IA, Vina JR (2006). Structure of the blood-brain barrier and its role in the transport of amino acids. J Nut.

[CR48] Herzog E, Bellenchi GC, Gras C, Bernard V, Ravassard P, Bedet C, Gasnier B, Giros B, El Mestikawy S (2001). The existence of a second vesicular glutamate transporter specifies subpopulations of glutamatergic neurons. J Neurosci.

[CR49] Hilbig H, Bidmon HJ, Oppermann OT, Remmerbach T (2004). Influence of post-mortem delay and storage temperature on the immunohistochemical detection of antigens in the CNS of mice. Exp Toxicol Pathol.

[CR50] Hioki H, Fujiyama F, Taki K, Tomioka R, Furuta T, Tamamaki N, Kaneko T (2003). Differential distribution of vesicular glutamate transporters in the rat cerebellar cortex. Neurosci.

[CR51] Hisano S, Hoshi K, Ikeda Y, Maruyama D, Kanemoto M, Ichijo H, Kojima I, Takeda J, Nogami H (2000). Regional expression of a gene encoding a neuron-specific Na(+)-dependent inorganic phosphate cotransporter (DNPI) in the rat forebrain. Mol Brain Res.

[CR52] Igarashi T, Huang TT, Noble LJ (2001). Regional vulnerability after traumatic brain injury: gender differences in mice that overexpress human copper, zinc superoxide dismutase. Exp Neurol.

[CR53] Imai Y, Ibata I, Ito D, Ohsawa K, Kohsaka S (1996). A novel gene iba1 in the major histocompatibility complex class III region encoding an EF hand protein expressed in a monocytic lineage. Biochem Biophys Res Commun.

[CR54] Jenkins WJ (1953). The significance of blood and cerebrospinal fluid urea levels estimated after death. J Clin Pathol.

[CR55] Jope RS, Jenden DJ (1979). Choline and phospholipid-metabolism and the synthesis of acetylcholine in rat-brain. J Neurosci Res.

[CR56] Kabadi SV, Stoica BA, Loane DJ, Byrnes KR, Hanscom M, Cabatbat RM, Tan MT, Faden AI (2012). Cyclin D1 gene ablation confers neuroprotection in traumatic brain injury. J Neurotrauma.

[CR57] Kaneko T, Fujiyama F (2002). Complementary distribution of vesicular glutamate transporters in the central nervous system. Neurosci Res.

[CR58] Kaneko T, Fujiyama F, Hioki H (2002). Immunohistochemical localization of candidates for vesicular glutamate transporters in the rat brain. J Comp Neurol.

[CR59] Kanno T, Nishizaki T, Proia RL, Kajimoto T, Jahangeer S, Okada T, Nakamura S (2010). Regulation of synaptic strength by sphingosine 1-phosphate in the hippocampus. Neurosci.

[CR60] Karam DW (2012) Neuroscience: a medical student’s guide. 1st edn. Trafford Publishing, EEUU

[CR61] Kind T, Wohlgemuth G, Lee DY, Lu Y, Palazoglu M, Shahbaz S, Fiehn O (2009). FiehnLib: mass spectral and retention index libraries for Metabolomics based on quadrupole and time-of-flight gas chromatography/mass spectrometry. Anal Chem.

[CR62] Klein J (2000). Membrane breakdown in acute and chronic neurodegeneration: focus on choline-containing phospholipids. J Neural Transm.

[CR63] Koleske AJ (2013). Molecular mechanisms of dendrite stability. Nat Rev Neurosci.

[CR64] Kosaka T, Heizmann CW (1989). Selective staining of a population of parvalbumin-containing GABAergic neurons in the rat cerebral cortex by lectins with specific affinity for terminal N-acetylgalactosamine. Brain Res.

[CR65] Kronke M (1999). Biophysics of ceramide signaling: interaction with proteins and phase transition of membranes. Chem Phys Lipids.

[CR66] Krzywinski M, Altman N (2014) Points of significance: Analysis of variance and blocking. Nat Meth 11(7):699–700. doi:10.1038/nmeth.3005. http://www.nature.com/nmeth/journal/v11/n7/abs/nmeth.3005.html#supplementary-information 10.1038/nmeth.3005 25110779

[CR67] Lavenex P, Lavenex PB, Bennett JL, Amaral DG (2009). Postmortem changes in the neuroanatomical characteristics of the primate brain: hippocampal formation. J Comp Neurol.

[CR68] Lavezzi AM, Corna MF, Matturri L (2013). Neuronal nuclear antigen (NeuN): a useful marker of neuronal immaturity in sudden unexplained perinatal death. J Neurol Sci.

[CR69] Lewis KE, Rasmussen AL, Bennett W, King A, West AK, Chung RS, Chuah MI (2014). Microglia and motor neurons during disease progression in the SOD1(G93A) mouse model of amyotrophic lateral sclerosis: changes in arginase1 and inducible nitric oxide synthase. J Neuroinflamm.

[CR70] Lindqvist D, Mueller S, Mellon SH, Su Y, Epel ES, Reus VI, Rosser R, Mahan L, Mackin RS, Yang TT, Wolkowitz OM (2014). Peripheral antioxidant markers are associated with total hippocampal and CA3/dentate gyrus volume in MDD and healthy controls-preliminary findings. Psychiat Res-Neuroim.

[CR71] Maccarrone M, Valverde O, Barbaccia ML, Castane A, Maldonado R, Ledent C, Parmentier M, Finazzi-Agro A (2002). Age-related changes of anandamide metabolism in CB1 cannabinoid receptor knockout mice: correlation with behaviour. Eur J Neurosci.

[CR72] Maskey D, Pradhan J, Oh CK, Kim MJ (2012). Changes in the distribution of calbindin D28-k, parvalbumin, and calretinin in the hippocampus of the circling mouse. Brain Res.

[CR73] Merino-Serrais P, Benavides-Piccione R, Blazquez-Llorca L, Kastanauskaite A, Rabano A, Avila J, DeFelipe J (2013). The influence of phospho-tau on dendritic spines of cortical pyramidal neurons in patients with Alzheimer’s disease. Brain.

[CR74] Mikuni N, Babb TL, Chakravarty DN, Chung CK (1998). Postnatal expressions of non-phosphorylated and phosphorylated neurofilament proteins in the rat hippocampus and the Timm-stained mossy fiber pathway. Brain Res.

[CR75] Minelli A, Alonso-Nanclares L, Edwards RH, Defelipe J, Conti F (2003). Postnatal development of the vesicular GABA transporter in rat cerebral cortex. Neurosci.

[CR76] Miyazaki T, Fukaya M, Shimizu H, Watanabe M (2003). Subtype switching of vesicular glutamate transporters at parallel fibre-Purkinje cell synapses in developing mouse cerebellum. Eur J Neurosci.

[CR77] Morris SM (2006). Arginine: beyond protein. Am J Clin Nutr.

[CR78] Morris SM (2007). Arginine metabolism: boundaries of our knowledge. J Nutr.

[CR79] Morrison LD, Becker L, Ang LC, Kish SJ (1995). Polyamines in human brain—regional distribution and influence of aging. J Neurochem.

[CR80] Mullen RJ, Buck CR, Smith AM (1992). NeuN, a neuronal specific nuclear protein in vertebrates. Development.

[CR81] Nakamura K, Hioki H, Fujiyama F, Kaneko T (2005). Postnatal changes of vesicular glutamate transporter (VGluT)1 and VGluT2 immunoreactivities and their colocalization in the mouse forebrain. J Comp Neurol.

[CR82] Natarajan V, Schmid PC, Schmid HHO (1986). N-acylethanolamine phospholipid-metabolism in normal and ischemic rat-brain. Biochim Biophys Acta.

[CR83] Naz S, Garcia A, Barbas C (2013). Multiplatform Analytical Methodology for Metabolic Fingerprinting of Lung Tissue. Anal Chem.

[CR84] Ni BH, Wu X, Yan GM, Wang J, Paul SN (1995). Regional expression and cellular localization of the Na(+)-dependent inorganic phosphate cotransporter of rat brain. J Neurosci.

[CR85] Nilsson G (1993). Surviving hypoxia, mechanisms of control and adaptation.

[CR86] Ohkubo T, Tanaka Y (2010). Administration of DHA-PS to aged mice was suitable for increasing hippocampal PS and DHA ratio. J Oleo Sci.

[CR87] Palkovits M, Harvey-White J, Liu J, Kovacs ZS, Bobest M, Lovas G, Bago AG, Kunos G (2008). Regional distribution and effects of postmortal delay on endocannabinoid content of the human brain. Neuroscience.

[CR88] Paxinos G, Franklin K (2001). The Mouse Brain in Stereotaxic Coordinates.

[CR89] Pearce RKB, Owen A, Daniel S, Jenner P, Marsden CD (1997). Alterations in the distribution of glutathione in the substantia nigra in Parkinson’s disease. J Neural Transm.

[CR90] Perry TL, Hansen S, Gandham SS (1981). *Postmortem* changes of amino-compounds in human and rat-brain. J Neurochem.

[CR91] Piyanoya A, Lomazzo E, Bindila L, Lerner R, Albayram O, Ruhl T, Lutz B, Zimmer A, Bilkei-Gorzo A (2015). Age-related changes in the endocannabinoid system in the mouse hippocampus. Mech Ageing Dev.

[CR92] Pocock JM, Kettenmann H (2007). Neurotransmitter receptors on microglia. Trends Neurosci.

[CR93] Ravid R, Swaab DF (1993). The Netherlands brain bank–a clinico-pathological link in aging and dementia research. J Neural Transm Suppl.

[CR94] Rowe CE (1969). Measurement of triglyceride in brain and metabolism of brain triglyceride *in vitro*. J Neurochem.

[CR95] Sarnat HB, Nochlin D, Born DE (1998). Neuronal nuclear antigen (NeuN): a marker of neuronal maturation in early human fetal nervous system. Brain Dev.

[CR96] Schmid PC, Krebsbach RJ, Perry SR, Dettmer TM, Maasson JL, Schmid HH (1995). Occurrence and postmortem generation of anandamide and other long-chain N-acylethanolamines in mammalian brain. FEBS Lett.

[CR97] Schmidt-Glenewinkel T, Nomura Y, Giacobini E (1977). The conversion of lysine into piperidine, cadaverine, and pipecolic acid in the brain and other organs of the mouse. Neurochem Res.

[CR98] Seiler N (2000). Oxidation of polyamines and brain injury. Neurochem Res.

[CR99] Simmons ML, Frondoza CG, Coyle JT (1991). Immunocytochemical localization of N-acetyl-aspartate with monoclonal-antibodies. Neuroscience.

[CR100] Smith RR, White HB (1968). Neutral lipid patterns of normal and pathologic nervous tissue—studies by thin layer chromatography. Arch Neurol.

[CR101] Soltys Z, Ziaja M, Pawlinski R, Setkowicz Z, Janeczko K (2001). Morphology of reactive microglia in the injured cerebral cortex. Fractal analysis and complementary quantitative methods. J Neurosci Res.

[CR102] Sorra KE, Harris KM (2000). Overview on the structure, composition, function, development, and plasticity of hippocampal dendritic spines. Hippocampus.

[CR103] Spokes EG (1979). An analysis of factors influencing measurements of dopamine, noradrenaline, glutamate decarboxylase and choline acetylase in human post-mortem brain tissue. Brain.

[CR120] Stepitak M, Dolezalo H (1974). Cadaverine in the brain of axenic mice. Nature.

[CR104] Stepita-Klauco M, Dolezalova H (1974). Cadaverine in the brain of axenic mice. Nature.

[CR105] Surendran S, Bhatnagar M (2011). Upregulation of N-acetylaspartic acid induces oxidative stress to contribute in disease pathophysiology. Int J Neurosci.

[CR106] Takamori S (2006). VGLUTs: ‘exciting’ times for glutamatergic research?. Neurosci Res.

[CR107] Tamamaki N, Yanagawa Y, Tomioka R, Miyazaki J, Obata K, Kaneko T (2003). Green fluorescent protein expression and colocalization with calretinin, parvalbumin, and somatostatin in the GAD67-GFP knock-in mouse. J Comp Neurol.

[CR108] Tyson RL, Sutherland GR (1998). Labeling of N-acetylaspartate and N-acetylaspartylglutamate in rat neocortex, hippocampus and cerebellum from [1-13C]glucose. Neurosci Lett.

[CR109] Unal-Cevik I, Kilinc M, Gursoy-Ozdemir Y, Gurer G, Dalkara T (2004). Loss of NeuN immunoreactivity after cerebral ischemia does not indicate neuronal cell loss: a cautionary note. Brain Res.

[CR110] Uysal N, Tugyan K, Aksu I, Ozbal S, Ozdemir D, Dayi A, Gonenc S, Acikgoz O (2012). Age-related changes in apoptosis in rat hippocampus induced by oxidative stress. Biotech Histochem.

[CR111] Varoqui H, Schafer MKH, Zhu HM, Weihe E, Erickson JD (2002). Identification of the differentiation-associated Na+/P-I transporter as a novel vesicular glutamate transporter expressed in a distinct set of glutamatergic synapses. J Neurosci.

[CR112] Whiley L, Godzien J, Ruperez FJ, Legido-Quigley C, Barbas C (2012). In-vial dual extraction for direct LCMS analysis of plasma for comprehensive and highly reproducible metabolic fingerprinting. Anal Chem.

[CR113] Williams RS, Ferrante RJ, Caviness VS (1978). The Golgi rapid method in clinical neuropathology: the morphologic consequences of suboptimal fixation. J Neuropathol Exp Neurol.

[CR114] Wolf HK, Buslei R, Schmidt-Kastner R, Schmidt-Kastner PK, Pietsch T, Wiestler OD, Blumcke I (1996). NeuN: a useful neuronal marker for diagnostic histopathology. J Histochem Cytochem.

[CR115] Wyss M, Kaddurah-Daouk R (2000). Creatine and creatinine metabolism. Physiol Rev.

[CR116] Yan HD, Ishihara K, Serikawa T, Sasa M (2003). Activation by N-acetyl-l-aspartate of acutely dissociated hippocampal neurons in rats via metabotropic glutamate receptors. Epilepsia.

[CR117] Yu H, Yoo PK, Aguirre CC, Tsoa RW, Kern RM, Grody WW, Cederbaum SD, Iyer RK (2003). Widespread expression of arginase I in mouse tissues: Biochemical and physiological implications. J Histochem Cytochem.

[CR118] Zhan X, Kim C, Sharp FR (2008). Very brief focal ischemia simulating transient ischemic attacks (TIAs) can injure brain and induce Hsp70 protein. Brain Res.

