# Supplementary material for: Metabolomics and neuroanatomical evaluation of post-mortem changes in the hippocampus
Source: Brain Struct Funct. 2017 Mar 11;222(6):2831–53. doi: 10.1007/s00429-017-1375-5 (PMC5541081; doi:10.1007/s00429-017-1375-5)
Supplement: Supplementary file 1 — Supplementary material 1 (DOCX 5613 KB) [file 429_2017_1375_MOESM1_ESM.docx]

**Supplementary Information (SI)**

**Authors:** Carolina Gonzalez-Riano^1¶^, Silvia Tapia^2,3,4¶^ , Antonia García^1^, Alberto Muñoz^2,3,4,5^, Javier DeFelipe^2,3,4*^, Coral Barbas^1*^

**Addresses:** ^1^CEMBIO (Centre for Metabolomics and Bioanalysis), Facultad de Farmacia, Universidad CEU San Pablo, Campus Monteprincipe, Boadilla del Monte, 28668, Madrid; ^2^Laboratorio Cajal de Circuitos Corticales (CTB), Universidad Politécnica de Madrid; ^3^Instituto Cajal (CSIC); and ^4^Centro de Investigación Biomédica en Red sobre Enfermedades Neurodegenerativas (CIBERNED), ISCIII, Madrid, Spain. ^5^Department of Cell Biology, Complutense University, Madrid, Spain.

¶ contributed equally to this work.

* Corresponding authors

Coral Barbas

CEMBIO (Center for Metabolomics and Bioanalysis),

Facultad de Farmacia, Universidad CEU San Pablo,

Campus Monteprincipe,

Boadilla del Monte,

28668 Madrid

Spain

Tel: (+34) 913726409

e-mail: cbarbas@ceu.es

Javier DeFelipe

Instituto Cajal (CSIC)

Avenida Doctor Arce 37

28002 Madrid

Spain

Tel: (+34) 91 336 4639

e-mail: defelipe@cajal.csic.es

**Title: METABOLOMICS AND NEUROANATOMICAL EVALUATION OF *POST-MORTEM* CHANGES IN THE HIPPOCAMPUS**

**Table of content:**

Supplementary Figures: S1-S7

Supplementary Tables: Table S1

**Fig S1.** Comparison of means of different metabolites at different PT times in hippocampus.


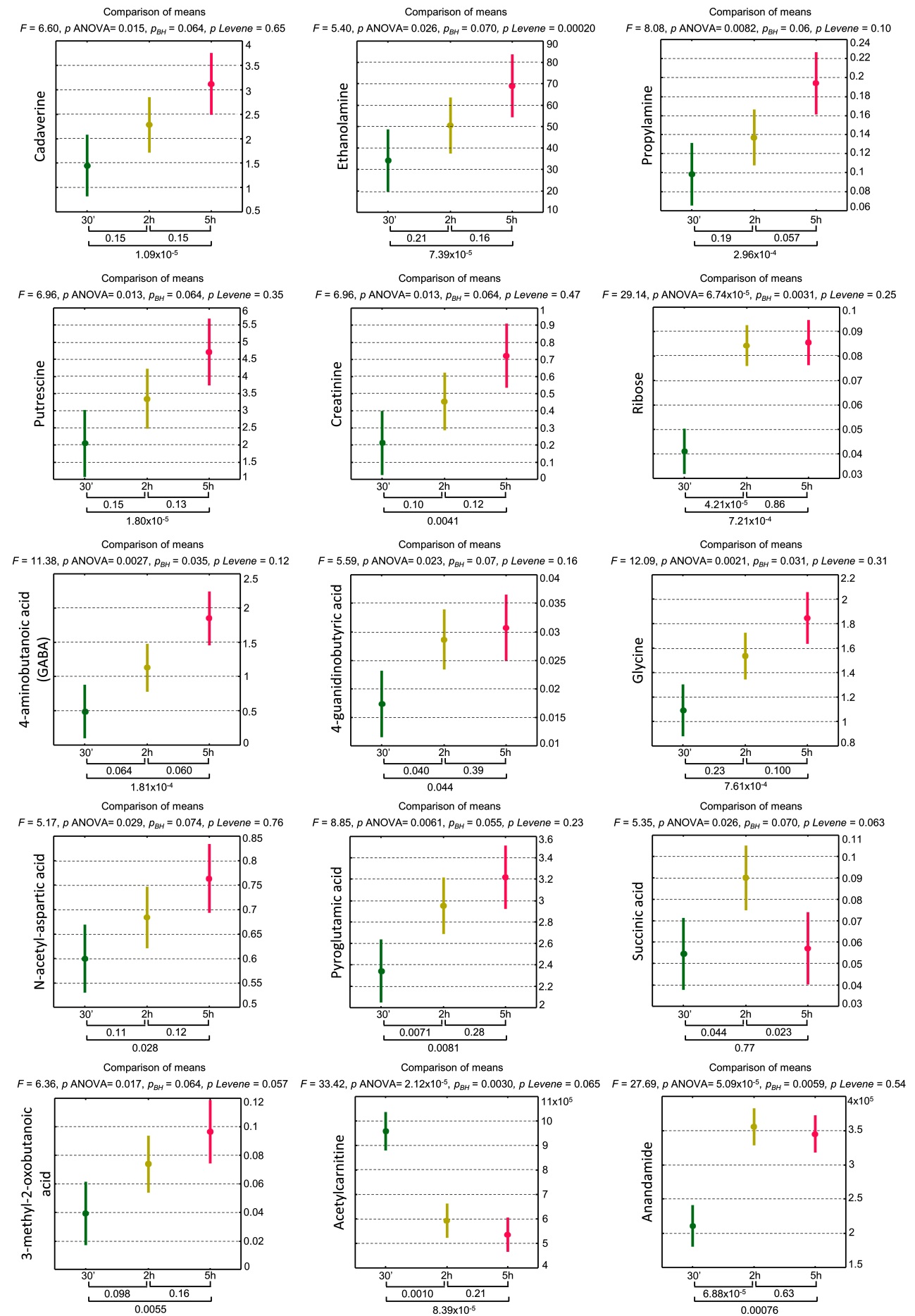


**Fig S1.** (continued 1).


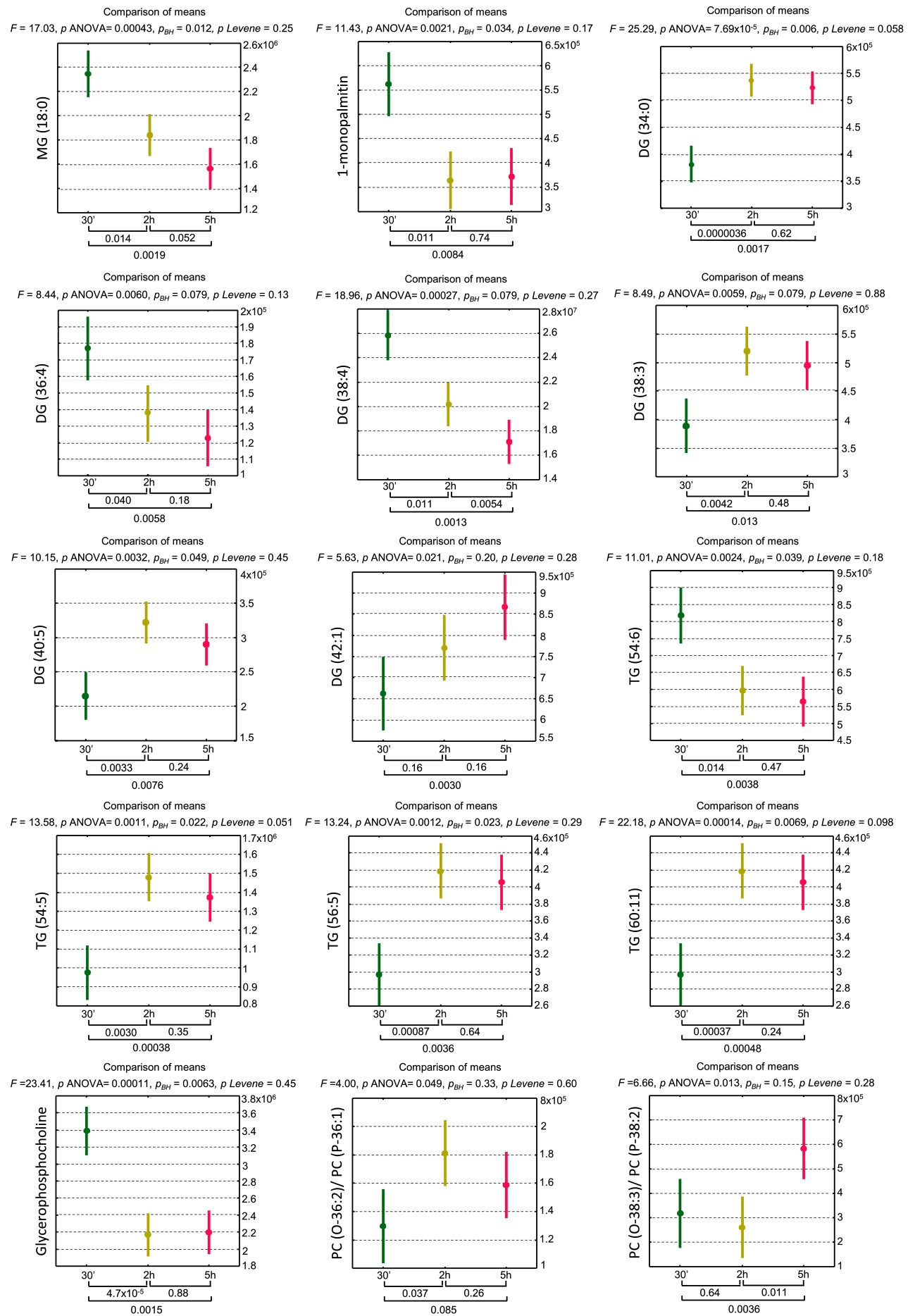


**Fig S1.** (continued 2).


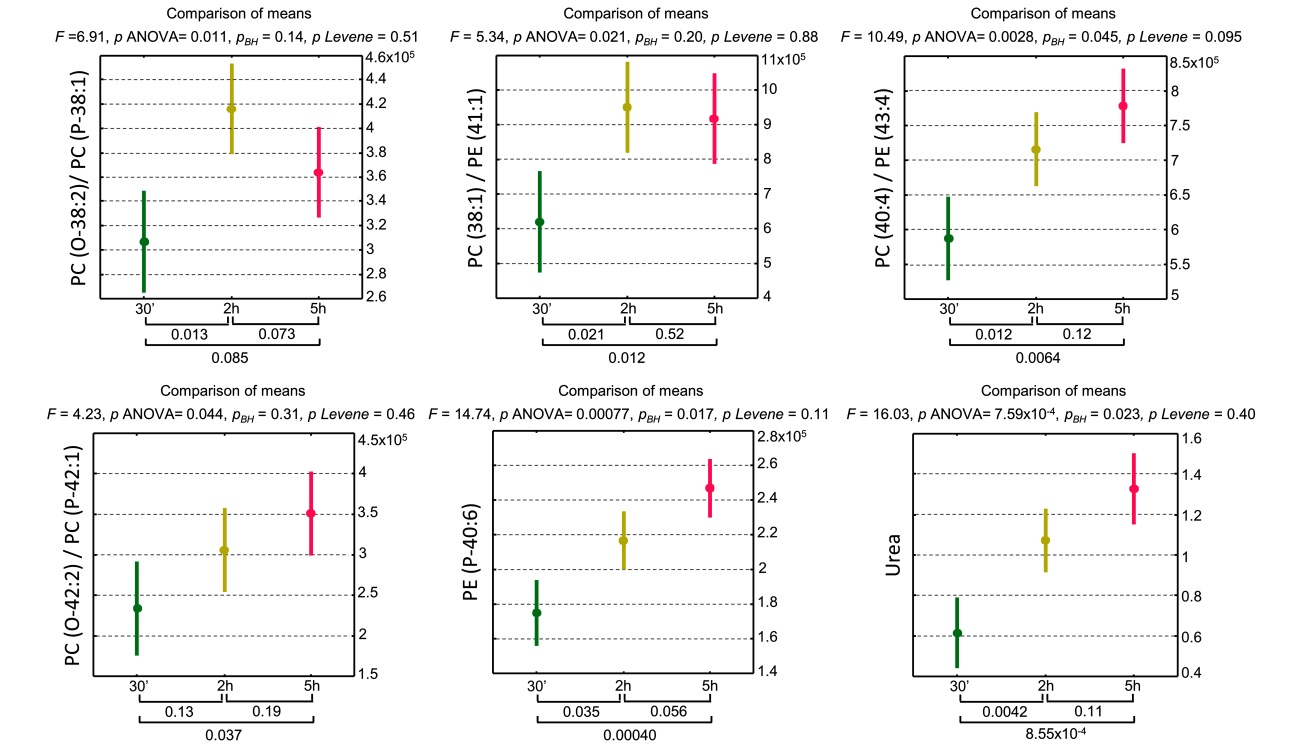


**Fig. S1**. Application of one-way ANOVA to a comparison of three groups. Sample means and their differences are represented by colored bars (green, 30-minute samples, 30’; yellow, 2-hour samples, 2h; pink, 5-hour samples, 5h). *p*-values for paired sample comparison were obtained by *t-*test. Statistic F, ANOVA *p*-value, corrected *p*-value (*p_BH_*), and Levene *p*-value are described on each graphic. Error bars, 95% confidence interval.

**
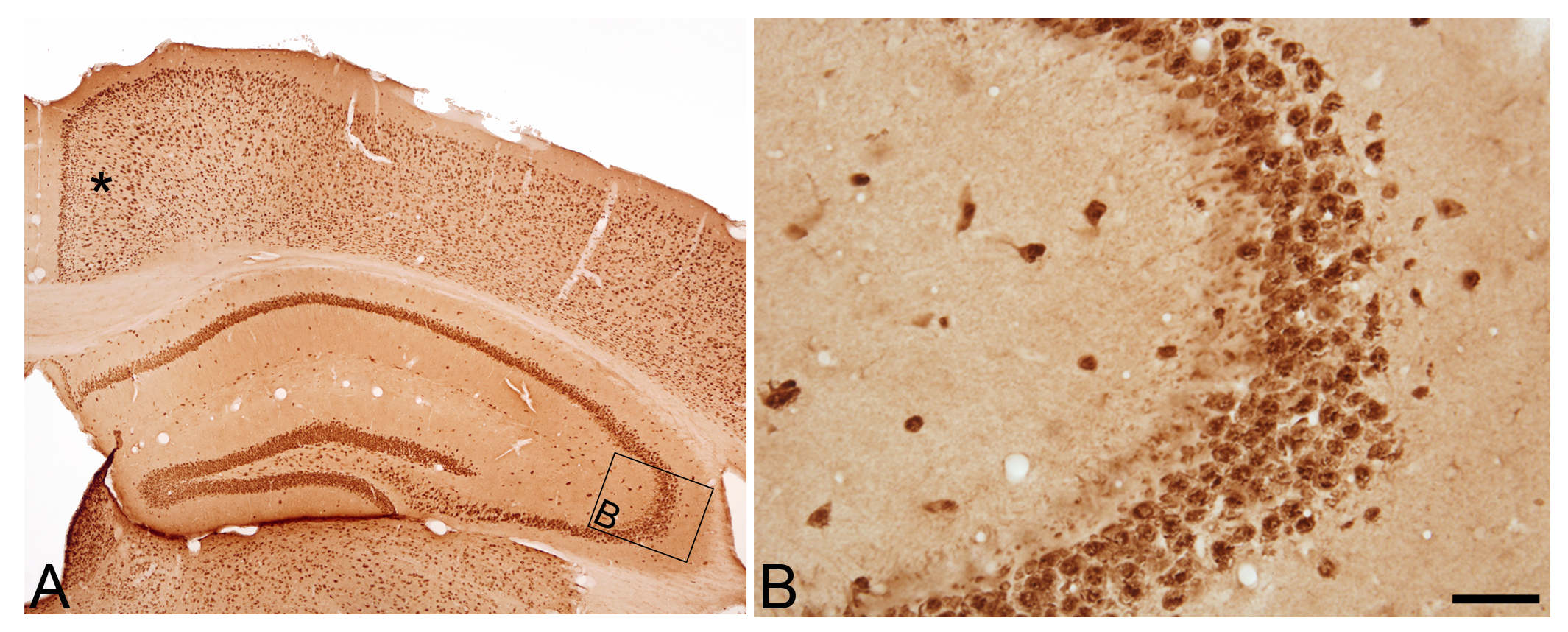
**

**Fig. S2**. NeuN-immunostaining in mouse cerebral cortex fixed by perfusion. **A**: Low-magnification photomicrograph showing NeuN immunostaining of a section from the neocortex and hippocampus of the brain of mouse fixed by perfusion. **B**: Higher magnification of the boxed area in A. Note that, in contrast to the images shown in Fig. 1 from animals fixed by immersion, there is an absence of zones that have a reduction in NeuN immunostaining both in the retrosplenial cortex (asterisk) and in the CA3 hippocampal region. However, in animals from 30 min PT onwards (see Fig. 1 D, E), but not at 0h PT (Fig. 1 C), there was a clear decrease in immunostaining in CA3. Scale bar (in B): 330 µm in A; 47µm in B.

**
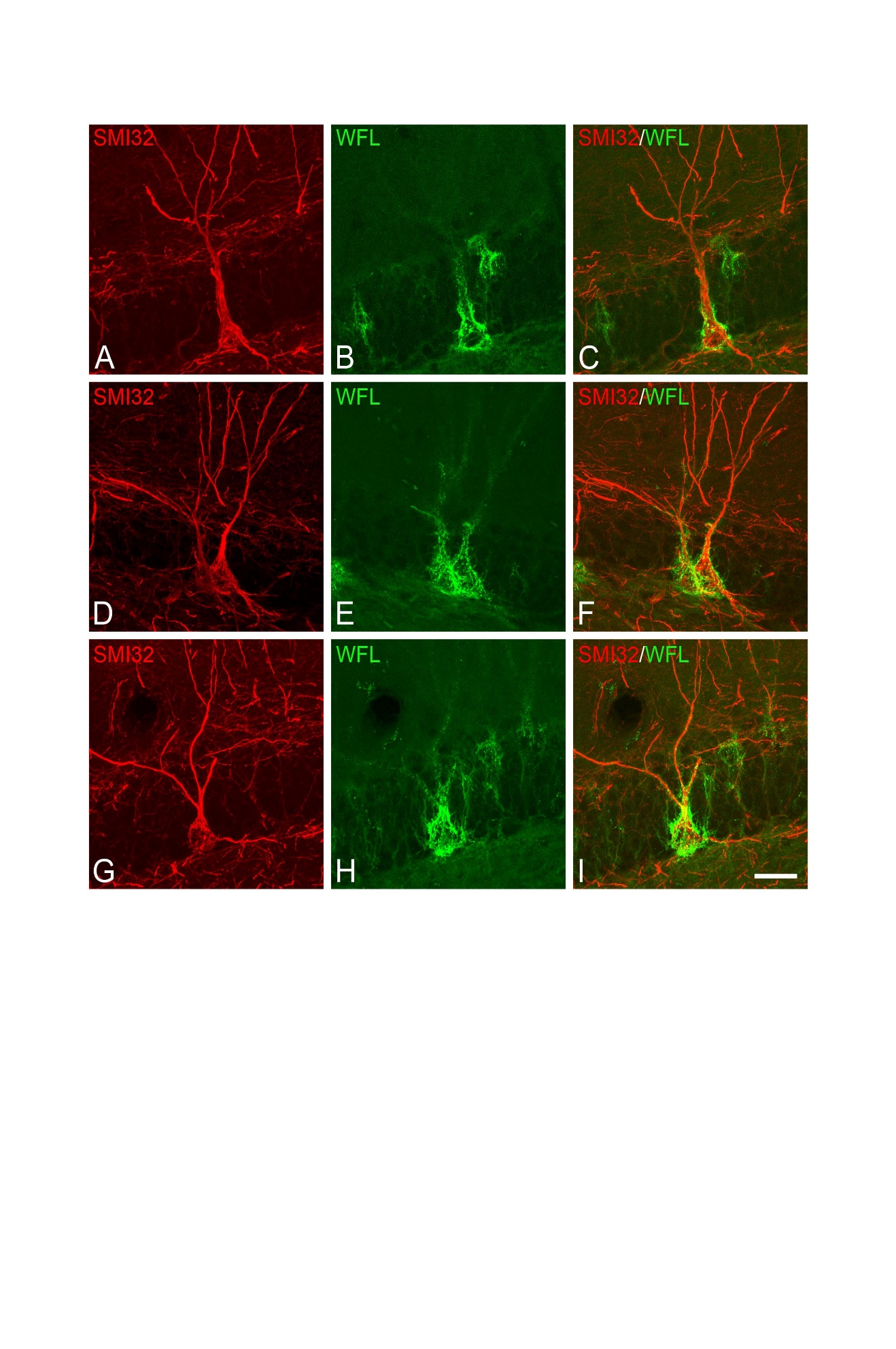
**

**Fig. S3**. Pairs of confocal images showing distribution of WFL and SMI-32 immunostaining in the CA1 field of the hippocampus of brains fixed by perfusion (A−C) or by immersion after 30 min (D−F) or 5 hours (G−I) PT. Note the similar pattern of SMI32 and WFL immunostaining of cell somata in perfusion and immersion fixed tissue. Scale bar (in I): 26 µm.


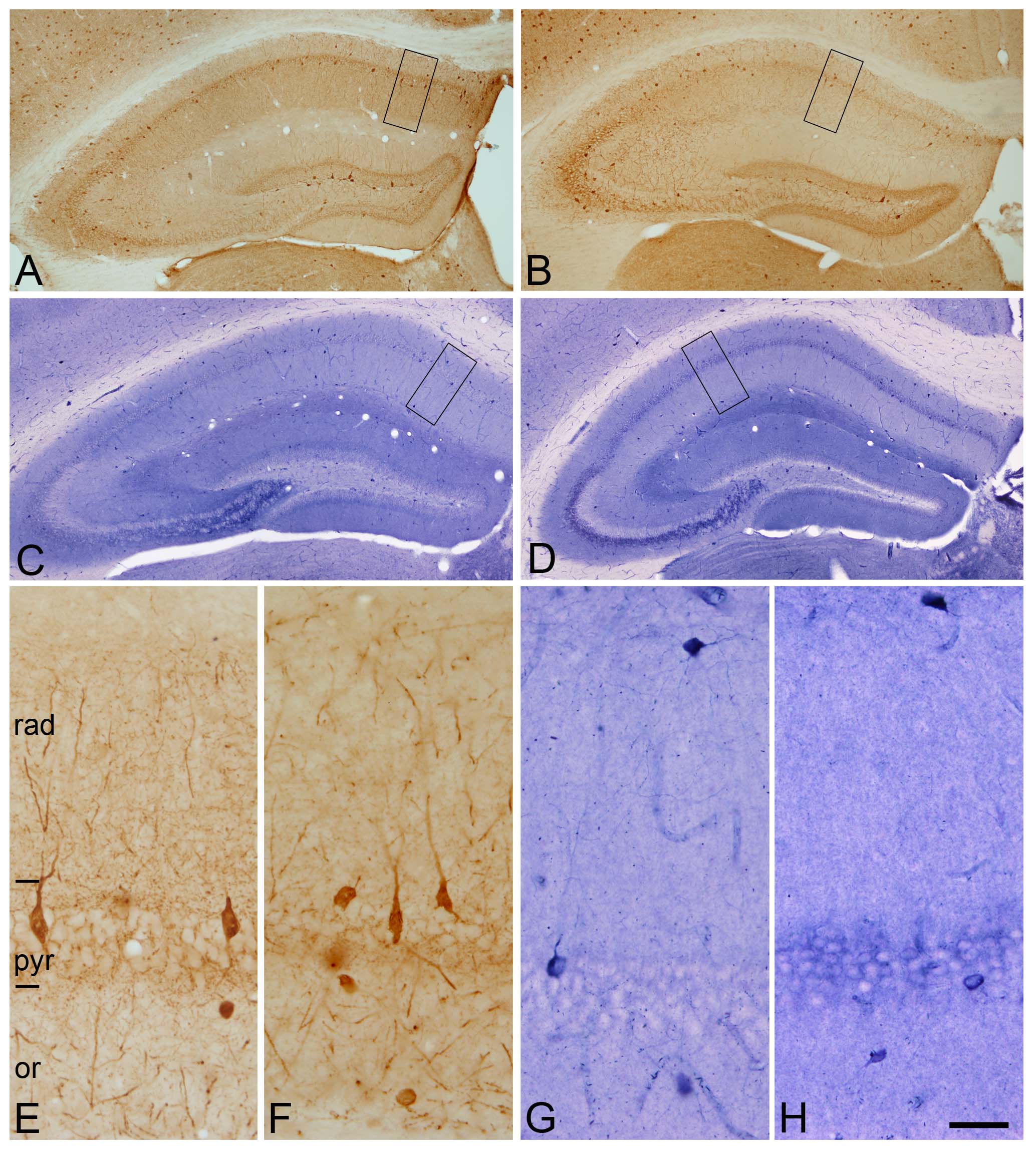


**Fig. S4**. Post-mortem time-related alterations in PV-immunostaining and histochemical staining for NADPH-d, in the mouse hippocampus. **A−D**: Photomicrographs showing the distribution patterns of PV-immunostaining (A, B, E and F) and NADPH-d-staining (C, D, G and H) in hippocampal sections of the brain of mice fixed by perfusion (A, C, E, G) as compared with those fixed by immersion after 5 hours PT (B, D, F, H). Note the marked changes that include increases and decreases in the labeling of elements (cell bodies, neuronal processes or puncta) depending on the hippocampal region and layer examined in immersion-fixed tissue as compared with tissue from perfused animals. For example, among other changes, there is a clear reduction in the labeling of PV-immunostaining processes and an increase in the NADPH-d staining in the stratum pyramidale of CA1 and CA3. **E−H**: High magnification photomicrographs showing the effect of PT on the PV-immunostaining and NADPH-d staining in CA1 region. Rectangles in A-D indicate the areas of magnification in E-H, respectively. or, stratum oriens; pyr, stratum pyramidale; rad, stratum radiatum. Scale bar: 275 µm in A−D; 35 µm E−H.


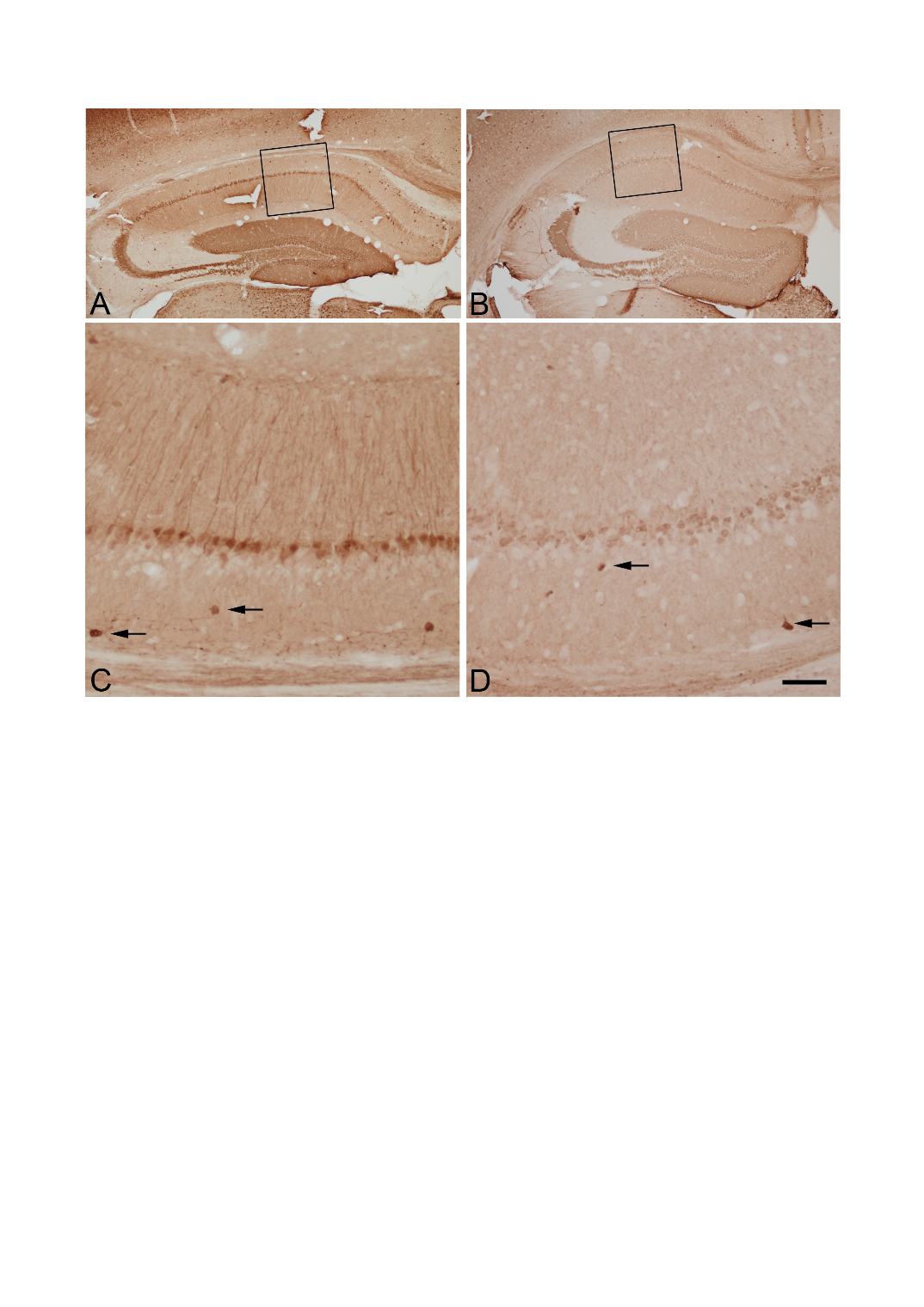


**Fig. S5.** Post-mortem time-related changes in CB-immunostaining in mouse hippocampus. Low (A, B)- and higher (C, D)-magnification photomicrographs of A and B, respectively (rectangles indicate the areas of magnification), showing CB-immunostaining of sections from hippocampus of the brain of mice fixed by perfusion (A, C) or by immersion after 5 hours PT (B, D). Note that the distribution pattern of CB- immunostaining in perfusion- and immersion-fixed tissue is similar but, in the latter case, there is a clear decrease in the CB-immunostaining. C, D illustrate the decrease in immunostaining in the somata and apical dendrites of CA1 pyramidal neurons. Scale bar: 300 µm in A, B; 50 µm in C, D.


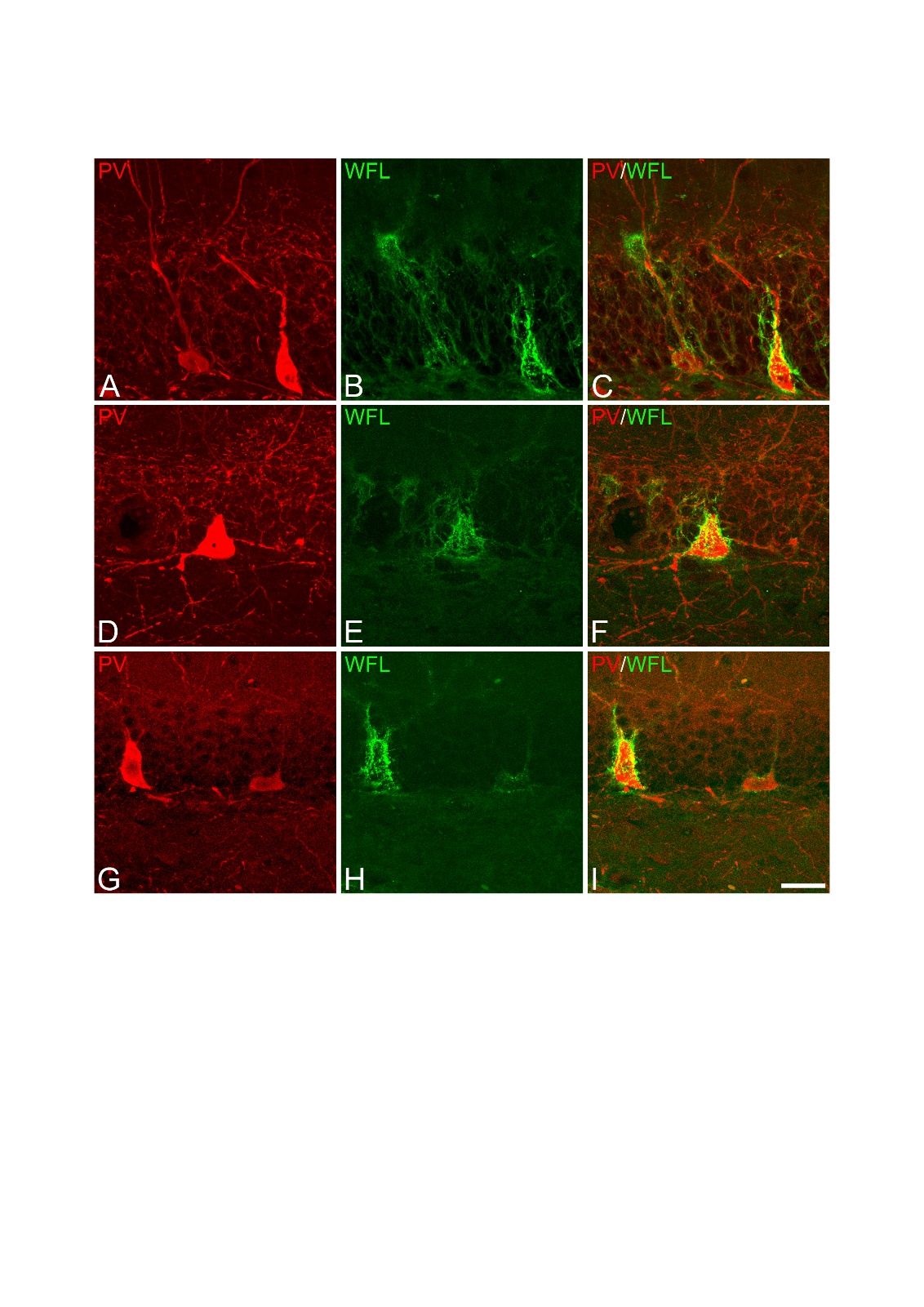


**Fig. S6**. Pairs of confocal images showing distribution of WFL and PV immunostaining in the dentate gyrus of brains fixed by perfusion (A−C) or by immersion after 30 min (D−F) or 5 hours (G−I) PT. Note the similar pattern of PV and WFL immunostaining of cell somata in perfusion and immersion fixed tissue. Scale bar (in I): 17 µm.


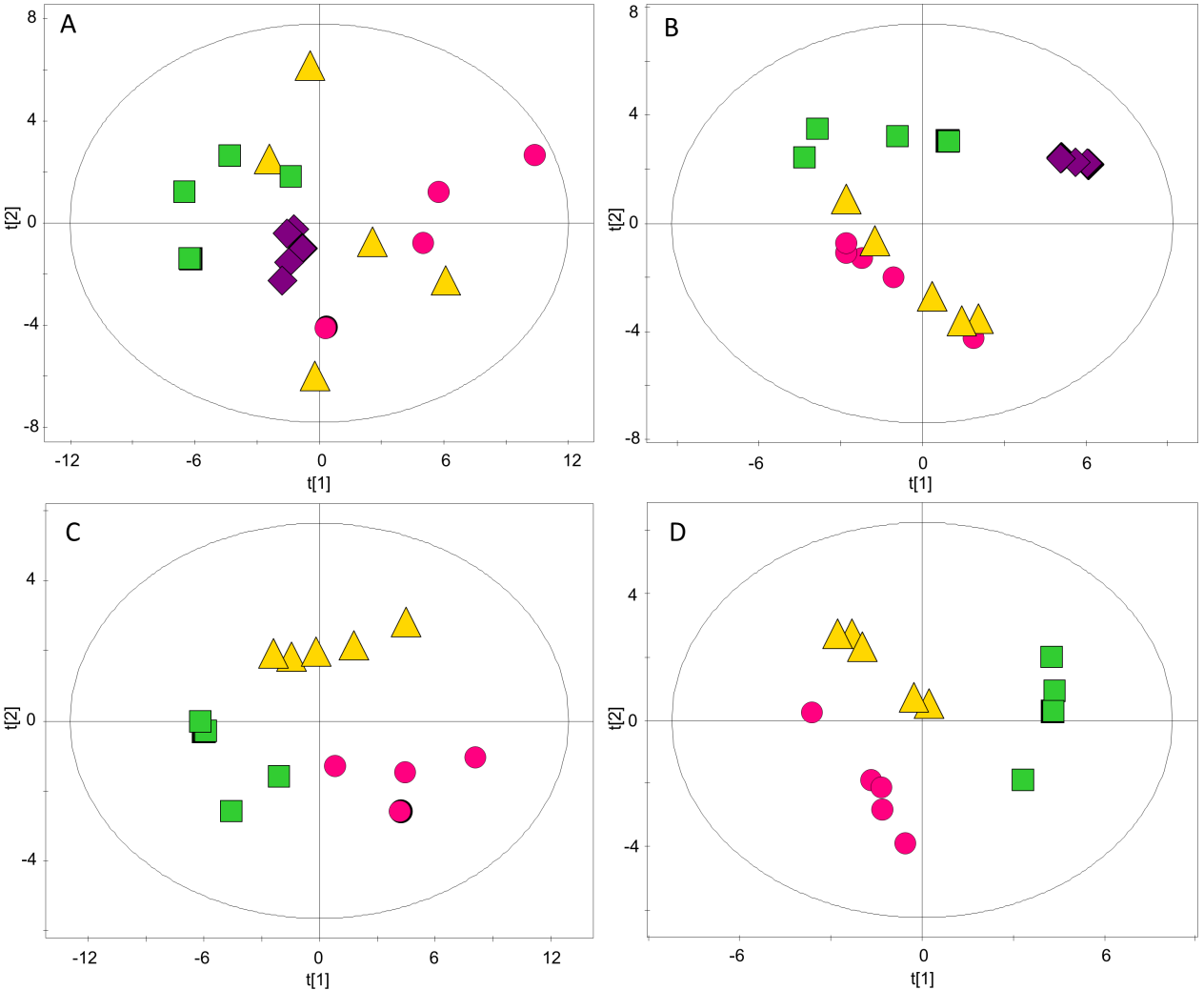


**Fig. S7**. PCA-X score plots for samples and QC samples (green squares, 30-minute samples, 30’; yellow triangles, 2-hour samples, 2h; pink dots, 5-hour samples, 5h; purple diamonds, QC samples). Plot A and plot B represent GC-MS and LC-MS analysis results, respectively. PLS-DA supervised models demonstrated a clear separation between the 3 groups. Plot C represents how samples are clustered in their respective groups for GC-MS analysis, with quality of variance explained and predicted variance (R^2^= 0.79; Q^2^ = 0.43); plot D shows the grouping of LC-MS samples (R^2^= 0.91; Q^2^ = 0.47).


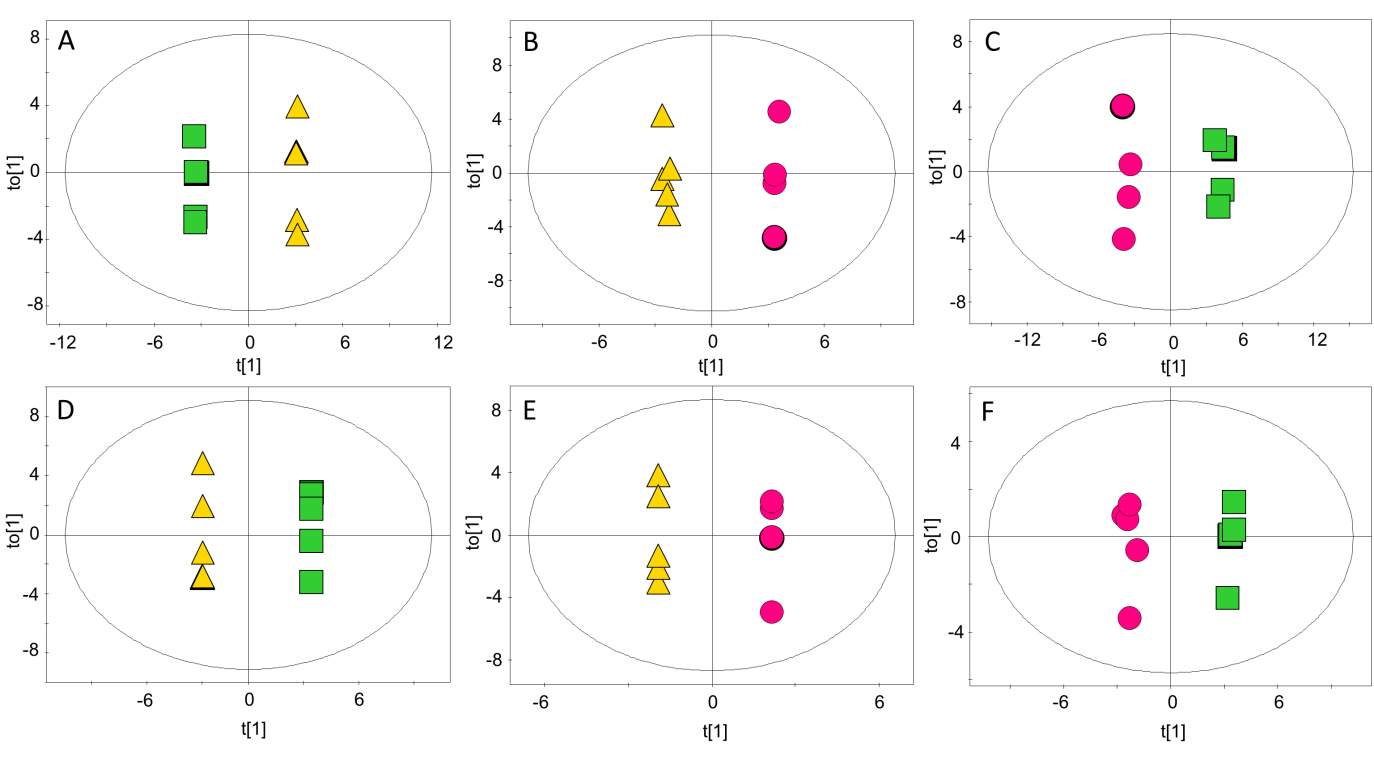


**Fig S8**. Supervised OPLS-DA models (green squares, 30-minute samples, 30’; yellow triangles, 2-hour samples, 2h; pink dots, 5-hour samples, 5h). Plots A−C represent GC-MS data in the following interpretation, 30’ *vs* 2h with quality of variance explained and predicted variance (R^2^ = 0.999, Q^2^ = 0.895), and percentage of samples correctly classified 80% ± 27.4 SD; 2h *vs* 5h (R^2^ = 0.997, Q^2^ = 0.824), and 90% ± 22.4 SD; 30’ *vs* 5h (R^2^ = 0.997, Q^2^ = 0.848), and 90% ± 22.4 SD. Plots D−F represent LC-MS data in the following interpretation, 30’ *vs* 2h with quality of variance explained and predicted variance (R^2^ = 0.999, Q^2^ = 0.931), and percentage of samples correctly classified 91.7% ± 20.4 SD; 2h *vs* 5h (R^2^ = 0.997, Q^2^ = 0.484), and 90% ±22.4 SD; 30’ *vs* 5h (R^2^ = 0.995, Q^2^ = 0.811), and 80% ± 27.4 SD.

**Table S1.** Summary of the primary antibodies used.

|  | | | | |
| --- | --- | --- | --- | --- |
| **Antigen** | **Immunogen** | **Source, host species, catalog number** | **Dilution used** | **Research Resource Identifiers (RRID)** |
| Anti-Calbindin D28K | Antiserum produced against recombinant rat calbindin-D28K | Swant. Rabbit polyclonal Cat#CB-38a | 1/2000 | AB_10000340 |
| Anti-GFAP (Glial Fibrillary Acidic Protein) | GFAP purified from human brain | Sigma-Aldrich, Rabbit polyclonal Cat#G9269 | 1/500 | AB_477035 |
| Anti-Glutamate Descarboxylase 65 (GAD65) | Human GAD65 from baculovirus infected cells | Millipore, Rabbit polyclonal Cat#AB5082 | 1/1000 | AB_2107925 |
| Anti-Glutamate Descarboxylase 65 (GAD65) | Synthetic peptide within Human GAD65 aa 541-567 conjugated to Keyhole Limpet Haemocyanin (KLH) | Abcam, Rabbit polyclonal Cat#ab203063 | 1/500 | - |
| Anti-Iba1 (Ionized calcium binding adaptor protein1) | Synthetic peptide corresponding to the Iba1 carboxy-terminal sequence | Wako, Rabbit polyclonal Cat#019-19741 | 1/500 | AB_2314666 |
| AntiNeuN | GST-tagged recombinant protein corresponding to mouse NeuN | Millipore, Rabbit policlonal, Cat#ABN78 | 1/2000 | AB_10807945 |

**Table S1** (continued).

| **Antigen** | **Immunogen** | **Source, host species, catalog number** | **Dilution used** | **Research Resource Identifiers (RRID)** |
| --- | --- | --- | --- | --- |
| Anti-Parvalbumin | Antiserum produced against rat muscle parvalbumin | Swant. Rabbit policlonal Cat#PV-25 | 1/2000 | AB_10000344 |
| Anti-SMI-32 | SMI-32 purified from ascites | Covance Research Products Inc., Mouse monoclonal Cat#SMI-32R-500 | 1/4000 | AB_10123763 |
| Anti-type 1 vesicular glutamate transporter | Synthetic peptide from rat VGLUT1 protein with no overlap to VGLUT2. The immunogen is available as AG208 | Millipore, Guinea pig polyclonal Cat#AB5905 | 1/5000 | AB_2301751 |
| Anti-type 2 vesicular glutamate transporter | Peptide corresponding to the C-terminal of rat VGLUT-2 | Millipore, Guinea pig polyclonal Cat#AB2251 | 1/2000 | AB_1587626 |
| Anti-vesicular GABA transporter, VIAAT | Synthetic peptide AEPPVEGDIHYQR (aa 75 - 87 in rat) coupled to key-hole limpet hemocyanin via an added N- terminal cysteine | Synaptic systems, Rabbit polyclonal Cat#131003 | 1/2000 | AB_887869 |
